# Impedance of Nonelectroneutral Solid Electrolyte Interphases With Nanopores: A Theoretical Model

**DOI:** 10.1002/advs.75601

**Published:** 2026-05-11

**Authors:** Chenkun Li, Jun Huang

**Affiliations:** ^1^ Institute of Energy and Climate Research IET‐3: Theory and Computation of Energy Materials Forschungszentrum Jülich GmbH Jülich Germany; ^2^ Faculty of Georesources and Materials Engineering RWTH Aachen University Aachen Germany

**Keywords:** constant‐phase element, impedance, nanopore, solid electrolyte interphase, space charge layer

## Abstract

Solid‐electrolyte interphases (SEI) in lithium batteries reportedly possess a charged solid‐solid interface and, in some cases, nanopores, while the effects of these two nonidealities on the impedance are ambiguous. Herein, we employ physical models to calculate local reaction conditions and resulting impedance spectra of nonelectroneutral and nanoporous SEIs under both nonreactive and reactive conditions. The calculated impedance is compared with existing experimental data. Under nonreactive conditions, low‐frequency constant‐phase element (CPE) phenomenon, which is prevalent in measurements yet remains puzzling, can be attributed to the nonelectroneutral local conditions in the SEI, because no CPE phenomenon is observed under electroneutral conditions. Under reactive conditions, the charge transfer resistance could grow, unexpectedly, with increasing overpotential during lithium stripping due to unfavorable local reaction environment. The structural parameters of nanopores within the inner layer markedly impact the impedance response, which cannot be captured by simple equivalent circuit models; physical models accounting for nanoconfined interfaces in the nanopores are needed, instead.

## Graphical abstract

Physical modeling reveals that often‐neglected non‐electroneutrality and nanopores in the solid‐electrolyte interphase (SEI) govern the impedance behavior. Under nonreactive conditions, the low‐frequency constant‐phase element (CPE) phenomenon can be attributed to the nonelectroneutral local conditions in the SEI. Under reactive conditions, the charge transfer resistance could grow, unexpectedly, with increasing overpotential during lithium stripping due to harsh local reaction environment. Nanopore structure strongly shapes impedance, beyond the predictive capability of simple equivalent circuit models, highlighting the need for physics‐based descriptions.

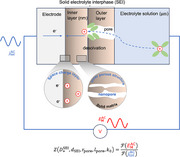

## 1 Introduction

### 1.1 Latest Understanding of the Solid‐Electrolyte Interphase

The solid‐electrolyte interphase (SEI) is a passivating layer formed on anode surfaces through electrolyte reduction [1, 2, 3] and the decomposition of anions, especially in high‐concentration and localized high‐concentration electrolyte, [4] during early cycling. The concept of the SEI was first coined by Peled in 1979 [5]. Long recognized as a key factor governing stability and degradation of lithium batteries, the SEI plays a dual role: it protects the electrode from continuous electrolyte decomposition while simultaneously consuming active lithium through ongoing formation and repair [6, 7, 8]. Although classical models—such as Peled's two‐layer model [6], the later mosaic model [7] and Aurbach's multi‐layered model [8]—have provided a foundational understanding of SEI, recent advances reveal their deficiencies, calling for a refined model.

**FIGURE 1:**
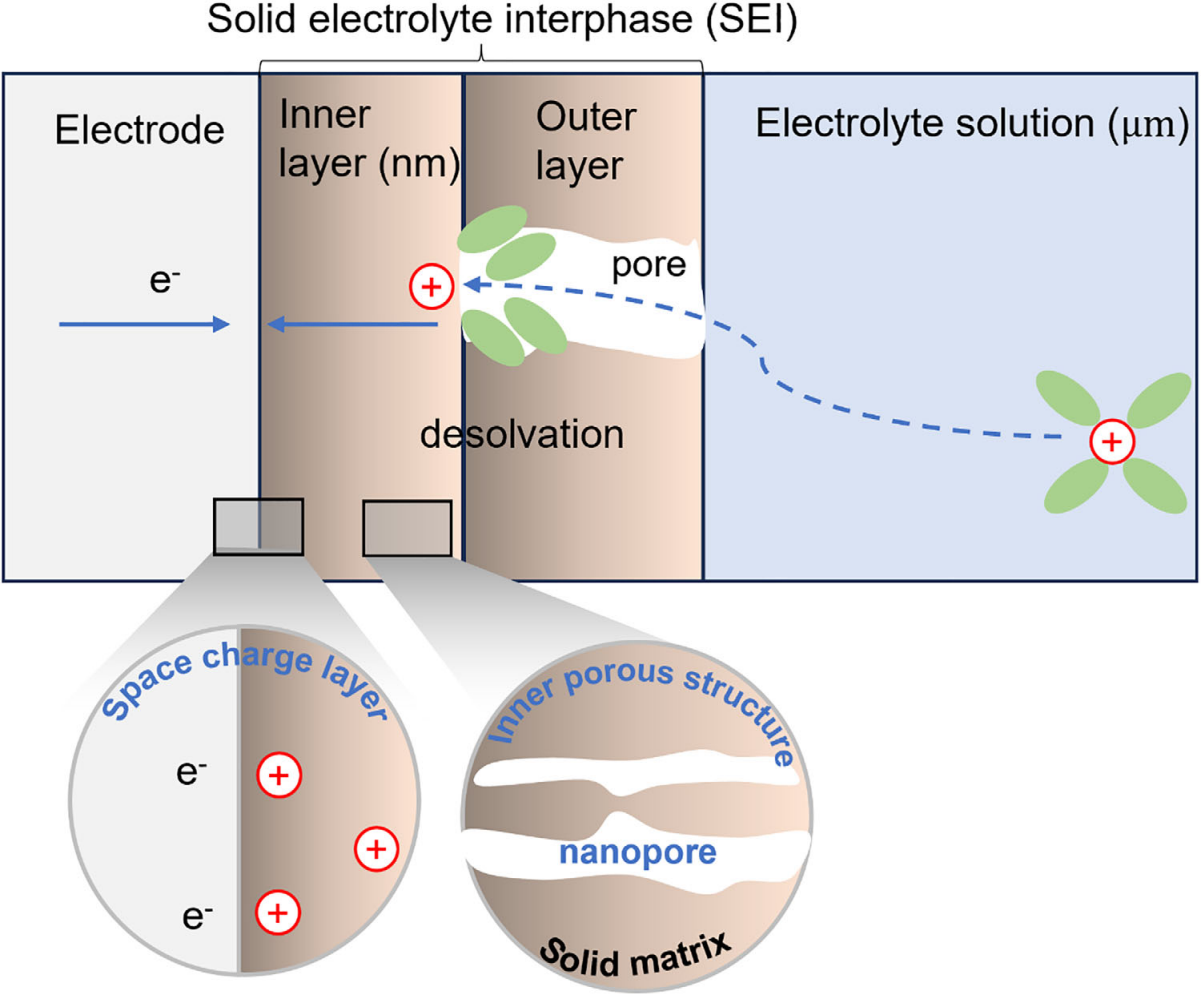
Schematic diagram of electrode‐SEI‐electrolyte solution interface with two new structural features, namely the space charge layer and nanopores that were not properly treated in existing physical models of SEI.

Recent studies have revealed two new features of the SEI as shown in Figure 1, namely, the space charge layer formed at the electrode‐SEI boundary and nanopores in the inner layer. Specifically, Qi et al. simulated the space charge layer formed at the electrode‐solid electrolyte interface using density functional theory (DFT) combined with Poisson‐Fermi‐Dirac equation, and determined interfacial distributions of electrons, defects and ions [9]. Yamamoto et al. experimentally probed the potential distribution at electrode‐solid electrolyte interface using quantitative electron holography [10]. They found a steep potential drop and a gradually extended slope caused by the space charge layer formed near the interface. Regarding the second new feature, the inner layer of the SEI is conventionally regarded as a compact phase without pores. Recently, Kranz et al. conducted a redox probe experiment and found that the diffusion of the redox molecules across pores in the inner SEI layer is faster than electron transport across the SEI, which suggested Li^+^ is primarily transported in the liquid electrolyte phase inside the pores of the inner layer [11]. Subsequently, Lv et al. used redox mediators of various sizes to probe the SEI layer formed in carbonate‐based electrolytes and also revealed the nanoporous structure of the inner layer of the SEI [12].

Unraveling the influence of space charge layer and nanopores on the properties of the SEI constitutes a pressing need. To this end, a variety of experimental techniques, including X‐ray photoelectron spectroscopy (XPS) [8, 13, 14], Fourier transform infrared spectroscopy (FTIR) [15, 16], nuclear magnetic resonance (NMR) [17, 18], and electrochemical impedance spectroscopy (EIS) [19, 20, 21, 22], as well as modeling approaches such as DFT [23], molecular dynamics (MD) [24, 25], ab initio molecular dynamics (AIMD) [26, 27], and kinetic Monte Carlo (KMC) [28, 29], have been employed. Among these methods, EIS stands out as a nondestructive, non‐invasive and operando method to investigate the formation, evolution and growth of the SEI [19, 20, 21, 22].

### 1.2 Previous Impedance Studies on SEI

Existing models for the impedance response of the SEI can be divided into two types: resistance‐capacitance/constant‐phase element (CPE) models and physical models. Specifically, the resistance‐capacitance/CPE model employs parallel resistance‐capacitance/CPE elements to describe both ion transport and capacitive response in the SEI, and it is widely used for fitting experimental data [30, 31, 32, 33]. The physical model uses underlying physical theories to describe ion migration and diffusion and interfacial charge transfer reactions [34, 35, 36]. In the following, we introduce recent progress of physical models of the SEI.

Latz et al. treated the SEI as a porous film and developed an approximate analytical solution under electroneutral conditions, namely, the electrode potential is exactly at the potential of zero charge (PZC) [34]. In their model, Li^+^ transport within the SEI was described by the diffusion equation with effective transport parameters. The SEI impedance (*Z*_SEI_) is expressed as a pure resistance (*R*_SEI_) in series with a finite‐length Warburg‐like transport impedance ($Z_{\mathrm{SEI}}^{\mathrm{Warg}}$), leading to $Z_{\mathrm{SEI}}=R_{\mathrm{SEI}}+Z_{\mathrm{SEI}}^{\mathrm{Warg}}$. They didn't obtain the impedance response under nonelectroneutral conditions. It is important to note that the electroneutral condition is an exception rather than the rule. In most scenarios, the inner layer of the SEI is a nonelectroneutral environment for charge transport, because the electrode potential is often not at the PZC.

Huang et al. extended Latz et al.’s work by considering a two‐layer SEI structure comprising an inner compact layer and an outer porous layer [35]. They derived an analytical solution for the SEI impedance also under electroneutral conditions. Li^+^ transport in the outer porous layer was described using the ambipolar diffusion equation with effective parameters. For the inner compact layer, they employed a phenomenological electric circuit model consisting of *R*_in_ and *C*_in_ in parallel to represent its impedance response. The Li^+^ desolvation at the compact‐porous interface was described using a Butler‐Volmer‐type equation.

Recently, Gaberscek et al. conducted a combined experimental and modeling research to clarify the physical origins of multiple semicircles observed in the SEI impedance [36]. They found that the mid‐frequency arc (“the SEI arc”) exhibits a weaker dependence on the electrolyte concentration compared to the ohmic and diffusion resistance. They further developed a 2D transmission line model considering the outer porous part of the SEI, accounting for the interactions between solid and liquid phase. However, the inner layer was considered to be fully compact, even though nanopores have been experimentally observed within it [11, 12]. Morasch et al. measured the SEI impedance on Cu and Ni foil electrodes in LP57 electrolyte (containing 2% VC) under blocking conditions [37]. They observed a high‐frequency semicircle followed by a low‐frequency tilt line. The high‐frequency semicircle was attributed to the coupled response of SEI resistance and capacitance, while the low‐frequency tilt line was assigned to double‐layer charging behavior. They further reported a potential‐dependent SEI resistance, which they interpreted as arising from changes in the dominant charge carriers (Li^+^) within the SEI. However, their analysis does not explicitly distinguish between possible contributions from inner versus outer SEI layers, nor does it consider the influence of a potentially porous microstructure on the impedance response.

Despite these advancements, no comprehensive framework has yet been developed to self‐consistently describe the nanoscopic SEI and the macroscopic electrolyte solution, and to calculate the impedance while accounting for the space‐charge layer at the electrode–inner‐layer interface and the nanopores within the inner layer. In addition, existing studies neglected the effect of the voltage bias, which is a general feature under operando conditions.

### 1.3 The Goal, Approach, and Outline of this Paper

Herein, we take a physics‐based approach to modeling the EIS of charged SEIs considering the space charge layer and the inner layer's nanopores, which are much less explored but highly relevant for experiments. Instead of using the ambipolar diffusion equation, we use Poisson‐Nernst‐Planck (PNP) equations to describe ion transport, releasing the electroneutrality assumption made in previous works. We aim at: (1) understanding the changes in EIS with electrode potential by correlating impedance with stationary distributions of ion concentration and electrostatic potential in the space charge layer inside the SEI; (2) exploring the influence of the structural factors of the SEI, especially, nanopores, on its EIS; (3) developing physics‐informed equivalent circuit models (ECM) to facilitate impedance‐based determination of structural and transport parameters of SEI under ideally blocking conditions; (4) understanding the impedance response of the SEI with lithium plating and stripping reactions. It should be highlighted that the SEI is highly heterogeneous, structurally dynamic, and challenging for in situ characterization. Current experimental data are generally insufficient to uniquely validate any model including the one to be developed here.

The reminder of this paper is organized as follows. First, we develop a 1D physical model to simulate the internal distributions and impedance response of the SEI under ideally blocking conditions. Second, we validate the numerical model under blocking conditions with experimental EIS data and provide insights into the CPE phenomenon in low‐frequency range. Third, we introduce lithium plating and stripping reactions and study the impedance response. Fourth, considering the possibility that realistic SEIs could be porous, we further build a 2D SEI model to study how the porous structure influences the impedance response under ideally polarizable conditions. Fifth, an analytical physics‐informed ECM is proposed to facilitate the analysis of experimental data. Sixth, we use this physics‐informed ECM to extract the SEI thickness, resistance, and capacitance over time from experimental data in literature. Finally, we summarize the influencing factors of SEI impedance.

## 2 Model Development

**FIGURE 2:**
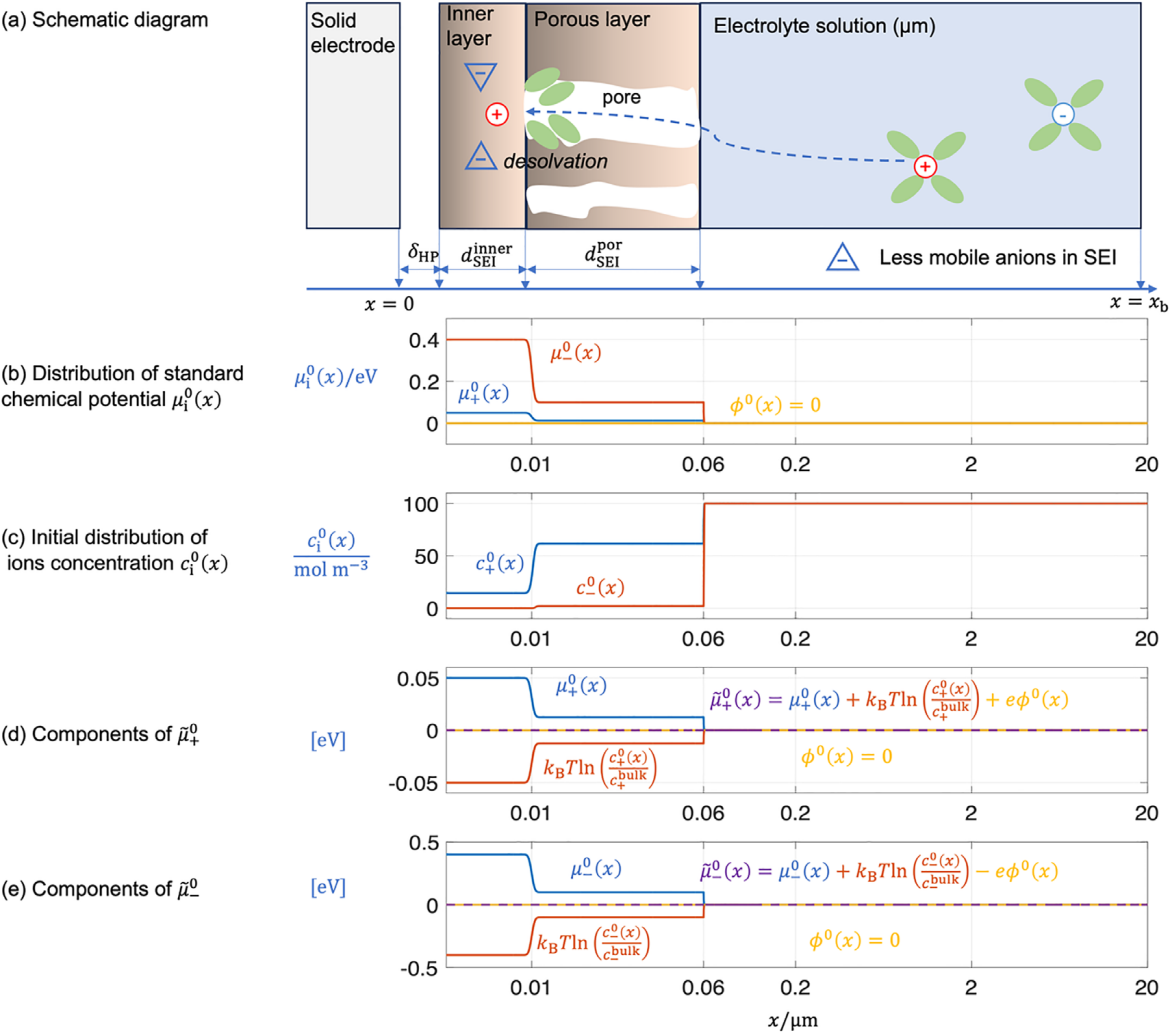
(a) Schematic illustration of the interfaces between the solid electrode, nanoscale SEI and electrolyte solution. δ_HP_ is the distance between the solid electrode and Helmholtz plane (HP), $d_{\mathrm{SEI}}^{\mathrm{inner}}$ and $d_{\mathrm{SEI}}^{\mathrm{por}}$ are the thicknesses of the inner layer and porous layer, respectively. (b) Distribution of standard chemical potential $\mu_{i}^{0}\left(x\right)$. (c) Initial distribution of ions concentration $c_{i}^{0}\left(x\right)$. (d) Distribution of components of electrochemical potential ${\tilde{\mu }}_{+}^{0}\left(x\right)$ of cations. (e) Distribution of components of electrochemical potential ${\tilde{\mu }}_{-}^{0}\left(x\right)$ of anions. Model parameters are as follows: $c_{\pm }^{\mathrm{bulk}}=0.1\;M$, $\mu_{-}^{0,\mathrm{inner}}=0.4\;\mathrm{eV},$ $\mu_{-}^{0,\mathrm{por}}=0.1\;\mathrm{eV},$ $\mu_{+}^{0,\mathrm{inner}}=0.05\;\mathrm{eV},$ $\mu_{+}^{0,\mathrm{por}}=0.0125\;\mathrm{eV}$, $d_{\mathrm{SEI}}^{\mathrm{inner}}=10\;\mathrm{nm}$, $d_{\mathrm{SEI}}^{\mathrm{por}}=50\;\mathrm{nm}$, *x*_b_ =  20 µm.

We consider a planar, two‐layer SEI located between an electrolyte solution and a solid electrode, as shown in Figure 2. The inner layer of the SEI is a compact phase while the outer layer has some mesopores filled with the electrolyte solution. The system is assumed to be homogeneous in the other two dimensions and thus treated in a 1D model focusing on the distributions in the thickness dimension. In the beginning, the solid electrode is assumed to be ideally polarizable. In EIS measurements, ideally polarizable conditions are often employed to eliminate possible interference of surface reactions on the ion transport. To approximate the ideally polarizable conditions, one can use copper (Cu), nickel (Ni), or gold (Au) as the working electrode (WE) immersed in an electrolyte solution composed of a lithium salt in an organic solvent. The electrode potential of the WE should be carefully tuned to lie in the range where possible Li^+^ deposition and intercalation reactions are minimal. Subsequently, we also consider the reactive case with lithium plating and stripping reactions.

Ion transport in the SEI and electrolyte solution is described uniformly using the Poisson‐Nernst‐Planck (PNP) equations [38, 39, 40],

(1) $$\frac{\partial c_{i}}{\partial t}+\nabla \;J_{i}=0$$

(2) $$-\nabla \;\left(\varepsilon \left(x\right)\nabla \phi \right)=F\sum_{i=\pm }z_{i}c_{i}-Fc_{\mathrm{back}}^{\mathrm{SEI}}\left(x\right)$$

with *J_i_* being the flux term expressed as,

(3) $$J_{i}=-\frac{D_{i}\left(x\right)}{k_{B}T}c_{i}\nabla \;{\tilde{\mu }}_{i}=-\frac{D_{i}\left(x\right)}{k_{B}T}c_{i}\nabla \left(\mu_{i}^{0}\left(x\right)+k_{B}T\ln\frac{c_{i}}{c_{i}^{\mathrm{bulk}}}+z_{i}e\phi \right)$$

where *i* denotes the cations (*i*  =   +) or anions (*i*  =   −), *z_i_* the charge number, $c_{i}/c_{i}^{\mathrm{bulk}}$ the concentration/reference concentration in the bulk electrolyte solution, *D_i_* (*x*) the diffusion coefficient depending on the transport mechanisms of Li^+^ in the inner layer of the SEI as detailed in the introduction section, *F* the Faraday constant, *R* the ideal gas constant, *T* the temperature, $c_{\mathrm{back}}^{\mathrm{SEI}}\left(x\right)$ is the concentration of immobile background charge inside the SEI, such as F^−^ in ref. [41], CO_3_^2−^ in ref. [42], and O^2−^ in ref. [43] etc., ensuring that the SEI is initially electroneutral and ϕ(x) is uniform,

(4) $$c_{\mathrm{back}}^{\mathrm{SEI}}\;\left(x\right)=\sum_{i=\pm }z_{i}c_{i}^{0}\left(x\right)\;$$

where $c_{i}^{0}\left(x\right)$ is the initial distribution of ion concentration.

Considering the difference in components and structure between the SEI and the electrolyte solution, we use a spatially distributed permittivity,

(5) $$\varepsilon \;\left(x\right)=\varepsilon_{\mathrm{SEI}}+\frac{\left(\varepsilon_{S}-\varepsilon_{\mathrm{SEI}}\right)}{2}\mathrm{erfc}\left(-\left(\frac{x-d_{\mathrm{SEI}}^{\mathrm{inner}}-d_{\mathrm{SEI}}^{\mathrm{por}}}{\lambda_{D}}\right)\right)$$

where ε_S_ is the permittivity of the electrolyte solution, ε_SEI_ the permittivity of the inner and porous layers of the SEI, $\lambda_{D}=\sqrt{\frac{\varepsilon_{S}RT}{F^{2}\sum c_{i}^{\mathrm{bulk}}}}$ the Debye length, and erfc is the complementary error function used to obtain a smooth transition of ε(*x*) from ε_SEI_ to ε_S_.

The standard chemical potential of species *i*, $\mu_{i}^{0}\left(x\right)$, is also spatially varying, as shown in Figure 2b. This reflects the fact that the ion concentration in the SEI is usually much lower than that in the electrolyte solution, e.g. $c_{+}^{\mathrm{SEI}}/c_{+}^{\mathrm{bulk}}\approx$1/100 in ref. [44] and $c_{+}^{\mathrm{SEI}}/c_{+}^{\mathrm{bulk}}\approx$1/1000–1/100 in ref. [45]. We note that the electrochemical potential of a species is uniform across the layers under equilibrium conditions,

(6) $${\tilde{\mu }}_{i}\;\left(x\right)=\mu_{i}^{0}\;\left(x\right)+k_{B}T\ln\left(\frac{c_{i}\left(x\right)}{c_{i}^{\mathrm{bulk}}}\right)+z_{i}e\phi \;\left(x\right)={{\tilde{\mu }}_{i}}^{\mathrm{bulk}}\;$$

and we set ${{\tilde{\mu }}_{i}}^{\mathrm{bulk}}=0$ as the reference value. Therefore, once we know the distribution of $\mu_{i}^{0}\left(x\right)$, the distribution of initial *c_i_* (*x*) can be obtained using Equation (6). We note that Colclasure et al. [45] and von Kolzenberg et al. [46] have suggested that $\mu_{i}^{0}\left(x\right)$ within the SEI is higher than that in the electrolyte solution. Different from Colclasure et al. [45] and von Kolzenberg et al. [46], we further incorporate the chemical potential of Li^+^ into transport equations.

Setting $\mu_{+}^{0,\mathrm{inner}}=0.05\;\mathrm{eV}$ and $\mu_{-}^{0,\mathrm{inner}}=0.4\;\mathrm{eV}$ inside the inner layer of the SEI, $\mu_{+}^{0,\mathrm{por}}=0.0125\;\mathrm{eV}$ and $\mu_{-}^{0,\mathrm{por}}=0.1\;\mathrm{eV}$ inside the porous layer of the SEI while zero in the electrolyte solution, we obtain the initial distribution of ion concentrations $c_{i}^{0}\left(x\right)$ as shown in Figure 2c. Since $\mu_{-}^{0}\left(x\right)>\mu_{+}^{0}\left(x\right)$ inside the SEI, anions are more difficult to enter into the SEI, resulting in $c_{-}^{0}\left(x\right)<c_{+}^{0}\left(x\right)$ inside the SEI. Figure 2d,e shows the distributions of components of electrochemical potential ${\tilde{\mu }}_{+}^{0}\left(x\right)$ and ${\tilde{\mu }}_{-}^{0}\left(x\right)$, respectively. We notice that initial equilibrium conditions ${\tilde{\mu }}_{+}^{0}\;\left(x\right)={\tilde{\mu }}_{-}^{0}\;\left(x\right)={{\tilde{\mu }}_{\pm }}^{\mathrm{bulk}}=0$ govern the trade‐off between the standard chemical potential $\mu_{i}^{0}\left(x\right)$ and the initial concentration distribution $c_{i}^{0}\left(x\right)$.

The PNP equations neglect the ion size effect, short range correlation, and solvent polarization. Extensions of PNP equations with one or several limitations released have been developed by Borukhov et al. [47], Gavish et al. [48, 49], Liu and Eisenberg [50], de Souza and Bazant [51], and also ourselves [52, 53]. We have compared the impedance responses calculated from the classical PNP equations and the modified PNP equations. The impedance shape remains unchanged, while moderate quantitative differences are observed (see Figure S1).

At the left boundary, which is located at the electrode‐inner layer of the SEI interface, *x*  = δ_HP_ , fluxes of both Li^+^ and anions are zero under the ideally blocking assumption,

(7) $$J_{Li^{+}}=0,\;J_{A^{-}}=0$$

where A^−^ denotes a monovalent anion such as ${\mathrm{PF}}_{6}^{-}$.

For the reactive case, namely, with lithium plating and stripping reactions Li^+^ + e↔Li occurring at the interface, the Li^+^ flux is correlated with the reaction current density,

(8) $$J_{Li^{+}}=\frac{j_{\mathrm{ct}}}{F}\;$$

where *j*_ct_ is the reaction current density described using the Frumkin‐corrected Butler‐Volmer equation [54], with the current density positive‐defined for the oxidation reaction following the International Union of Pure and Applied Chemists (IUPAC) convention [55],

(9) $$j_{\mathrm{ct}}=Fk_{0}\left(e^{\frac{\alpha F}{RT}\eta }-\frac{c_{+}^{\mathrm{HP}}}{c_{+}^{0}}e^{-\frac{\left(1-\alpha \right)F}{RT}\eta }\right)$$

where *k*_0_ is the reaction rate constant, α is the charge transfer coefficient, $c_{+}^{\mathrm{HP}}$ is the cation concentration at the HP and η is the overpotential defined as,

(10) $$\eta =E_{M}\;-\phi_{\mathrm{HP}}-E_{\mathrm{eq}}$$

with $\phi_{\mathrm{HP}}$ being the electrostatic potential at the HP and *E*_eq_ the equilibrium potential. Notably, $\phi_{\mathrm{HP}}$ is obtained directly from the numerical solution of the PNP equations. This treatment differs from the porous‐electrode framework developed by Biesheuvel and Bazant [56], as well as more recent asymptotic PNP formulations by Gupta et al. [57] and Janssen et al. [58], in which the electrical double layer is described using thin‐double‐layer asymptotics, such as Gouy–Chapman–Stern or modified Donnan models, under the assumption of quasi‐equilibrium within the double layer. Such formulations are appropriate when the Debye length is much smaller than the characteristic length scale of the system, allowing analytical reduction of the governing equations. In contrast, the present formulation does not assume a thin double layer, and therefore remains applicable in situations where the double‐layer thickness is comparable to the transport length scale or when the dynamic coupling between ion transport and interfacial kinetics must be resolved self‐consistently.

Here we consider there is a gap without space charge between the electrode surface and the HP. Therefore, the electric potential is linear in the space between the electrode surface and the HP [40, 59], leading to the following relationship between $\phi_{\mathrm{HP}}$ and *E*_M_,

(11) $$\phi_{\mathrm{HP}}=E_{M}\;-E_{\mathrm{pzc}}+\frac{\varepsilon_{S}\delta_{\mathrm{HP}}}{\varepsilon_{\mathrm{HP}}}\frac{\partial \phi }{\partial x}$$

where *E*_pzc_ is the potential of zero charge (PZC), ε_HP_ the dielectric permittivity in the space between the electrode surface and the HP, δ_HP_ the distance from the electrode surface to the HP, respectively. This boundary condition is different from $\phi =E_{M}$ widely used in the literature [60, 61, 62, 63], which neglects the gap between the electrode surface and HP, the potential drop therein, and the difference between the HP and the electrolyte solution in dielectric permittivity, which are crucial to the interfacial double‐layer structure and distributions of *c_i_* and ϕ [64]. Experimental observations have revealed an Equation (11)‐like distribution of the electrostatic potential in solid‐state batteries [10]. It is noted that *E*_M_, *E*_eq_, and *E*_pzc_ should adopt the same potential scale.

For the right boundary, which is located at the bulk solution, *x*  = *x*_b_ , all ions have their bulk concentrations, and the electric potential is set at zero as a reference, namely,

(12) $$\begin{array}{c} c_{Li^{+}}=c_{Li^{+}}^{\mathrm{bulk}},\;\;c_{A^{-}}=c_{A^{-}}^{\mathrm{bulk}}\\ \phi =0 \end{array}$$

The impedance *Z*_tot_ is defined as the ratio of Fourier‐transformed (F) electrode potential *E*_M_ over the tot current density *j*_tot_ [65],

(13) $$\begin{array}{c} Z_{\mathrm{tot}}=\frac{F\left(E_{M}\right)}{F\left(j_{\mathrm{tot}}\right)}\\ j_{\mathrm{tot}}=j_{\mathrm{ct}}+j_{\mathrm{dl}} \end{array}$$

with *j*_dl_ being the electrical double layer (EDL) current density defined as,

(14) $$\begin{array}{c} j_{\mathrm{dl}}=\frac{dQ_{M}}{dt}\\ Q_{M}=-\varepsilon_{\mathrm{HP}}\nabla \phi_{\mathrm{HP}} \end{array}$$

where *Q*_M_ is the free charge on the metal surface. There is another definition of the EDL charge calculated from the net charge in the electrolyte solution [66, 67], $Q_{\mathrm{sol}}=-F\int_{0}^{x_{b}}\sum_{i=\pm }z_{i}c_{i}dx$. *Q*_sol_ and *Q*_M_ are identical if the electric field vanishes at *x*  = *x*_b_  [68]. It is important to note that *Q*_sol_ neglects the dielectric response of the electrolyte solution, resulting in the absence of the dielectric capacitance as revealed in ref. [59]. If there is no charge transfer reaction, then we have *j*_ct_ =  0 and *j*_tot_ = *j*_dl_ .

**FIGURE 3:**
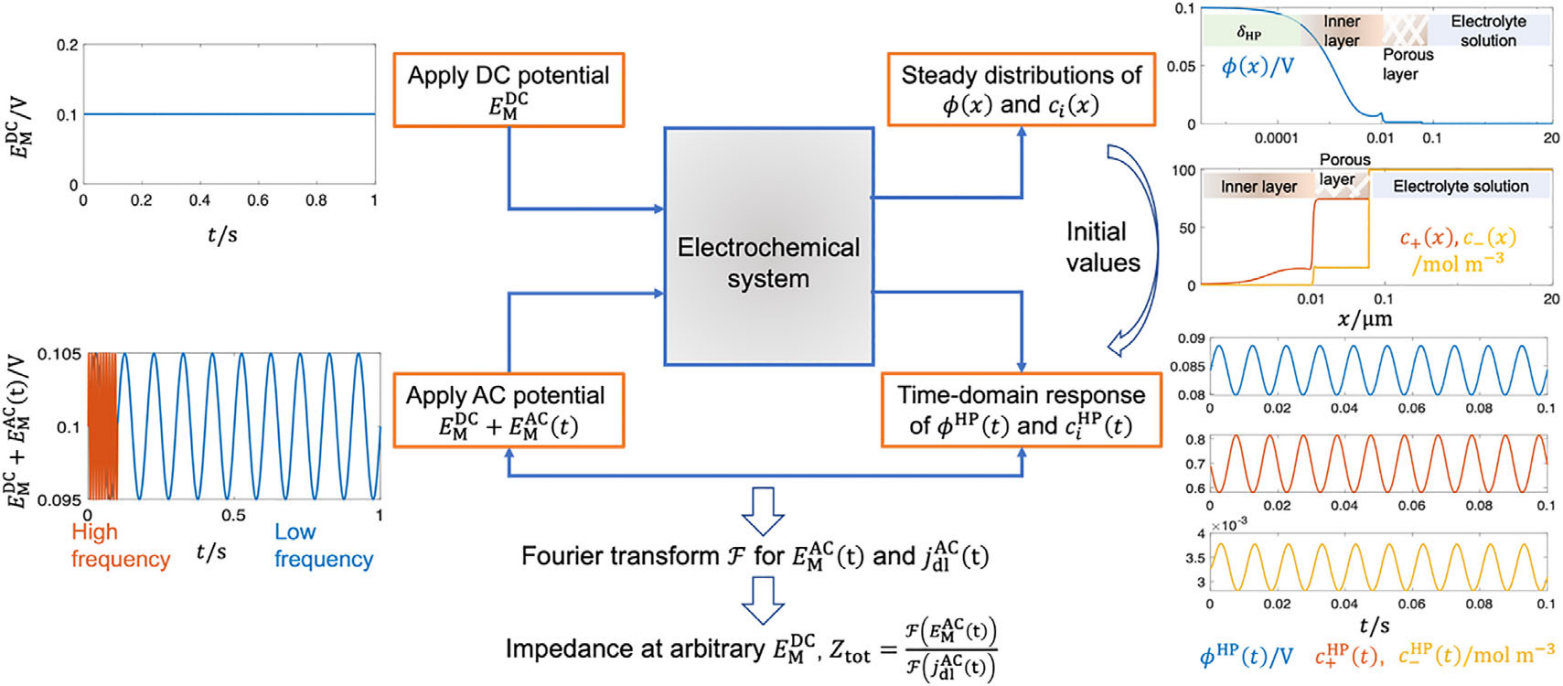
Numerical workflow of simulating the SEI impedance response to mimic the experimental protocol of EIS measurements. In the simulation, a DC electrode potential $E_{M}^{\mathrm{DC}}$ is first applied to obtain steady‐state distributions of ϕ(x) and *c_i_* (*x*). These distributions serve as initial values for the next step, where AC electrode potentials $E_{M}^{\mathrm{AC}}\left(t\right)$ with different frequencies are superimposed onto $E_{M}^{\mathrm{DC}}$. The resulting time‐dependent responses, $\phi^{\mathrm{HP}}\left(t\right),\;c_{+}^{\mathrm{HP}}\left(t\right)$ and $c_{-}^{\mathrm{HP}}\left(t\right)$, are then used to calculate the total impedance *Z*_tot_ via Equation (13).

The workflow of numerical solutions is shown in Figure 3, which mimics the experimental protocol of EIS measurements. In experiments, the system is typically rested at the open‐circuit voltage (OCV) or bias voltages for a sufficient duration to reach a steady state. Impedance is then measured by applying a small sinusoidal perturbation signal over a wide frequency range [69, 70, 71]. In numerical simulation, we closely mimic the experimental protocol. First, a DC electrode potential $E_{M}^{\mathrm{DC}}$ is applied to the model system to obtain the steady‐state distributions of ϕ(x) and *c_i_* (*x*). These distributions are then used as the initial values in the next step. Second, AC electrode potentials $E_{M}^{\mathrm{AC}}\left(t\right)$ with different frequencies are superimposed onto $E_{M}^{\mathrm{DC}}$ and applied to the system. We obtain $\phi^{\mathrm{HP}}\left(t\right),\;c_{+}^{\mathrm{HP}}\left(t\right)$ and $c_{-}^{\mathrm{HP}}\left(t\right)$ accordingly. Finally, we obtain the impedance *Z*_tot_ using Equation (13). While the electrode potential excitation is generally suitable, it tends to produce inaccurate results in the very low‐frequency region. To address this limitation, in the following subsection on the constant phase element phenomenon at charged SEI, we use current excitation as the input signal to calculate the impedance response.

## 3 Results

EIS is a linear response function of the electrochemical system around a steady state. First, to prepare the ground for understanding the simulated EIS, we calculate the steady distributions of $\phi \left(x\right),\;c_{+}\left(x\right)$ and *c*_−_(*x*) in the SEI for the two‐layer and inner‐layer SEI models at different porosities under ideally blocking conditions. Second, we compare the impedance response of the two‐layer and inner‐layer SEI models, and find that the SEI impedance is dominated by the inner compact layer. Therefore, we focus on the inner‐layer SEI model in the subsequent analysis. Third, we compare the steady distributions of $\phi \left(x\right),\;c_{+}\left(x\right)$ and *c*_−_(*x*) for the inner‐layer SEI model at different *E*_M_. Fourth, we calculate the EIS of the inner‐layer SEI model at these *E*_M_ values and give the time‐dependent $c_{+}^{\mathrm{HP}}\left(t\right)$ at different characteristic frequencies to aid in understanding the calculated EIS profiles. Fifth, we employ the numerical model to fit experimental data measured under blocking conditions at different *E*_M_. The model reveals the CPE phenomenon in the low‐frequency region. Sixth, we study the case with lithium plating and stripping reactions and calculate steady distributions of $\phi \left(x\right),\;c_{+}\left(x\right)$ and *c*_−_(*x*) and the impedance response. Finally, we build a 2D porous inner‐layer SEI model to study the effects of porous structures on the SEI impedance.

### 3.1 SEI Impedance Is Dominated by the Inner Compact Layer

The parameters used in model simulation are explained first. We divide the parameters into five groups: structural parameters including $d_{\mathrm{SEI}}^{\mathrm{inner}}$, $d_{\mathrm{SEI}}^{\mathrm{por}}$, ε_p_ and *x*_b_, material property parameters including $c_{\pm }^{\mathrm{bulk}}$, ε_SEI_, ε_S_ and $\mu_{\pm }^{0}$, kinetic parameters including $D_{\mathrm{SEI}}^{\mathrm{inner}}$, $D_{\mathrm{SEI}}^{\mathrm{por}}$ and *D*_bulk_, reaction parameters including α and *k*_0_, and measurement parameters including frequency and *E*_M_.

For the structural parameters, $d_{\mathrm{SEI}}^{\mathrm{inner}}$ denotes the thickness of the inner layer, ranging from 5 to 20 nm. $d_{\mathrm{SEI}}^{\mathrm{por}}$ is about ten nanometers to hundreds of nanometers [72]. The porosity ε_p_ is usually less than 0.1. *x*_b_ is a few micrometers to hundreds of micrometers [73]. Here we use $d_{\mathrm{SEI}}^{\mathrm{inner}}=10\;\mathrm{nm}$, $d_{\mathrm{SEI}}^{\mathrm{por}}=50\;\mathrm{nm}$, ε_p_ =  0.1 and *x*_b_ =  20 µm as basal values.

For the material property parameters, $c_{\pm }^{\mathrm{bulk}}$ can be varied from mm to m in experiments. ε_S_ is between 5ε_0_ and 20ε_0_ for common organic solvent used in lithium‐ion batteries [3]. ε_SEI_ is smaller than ε_S_ due to the strong interfacial electric field that restricts the orientation of solvent molecules near the electrode surface. Here we set $c_{\pm }^{\mathrm{bulk}}=0.1\;M$, ε_S_ =  13ε_0_ and ε_SEI_ =  6ε_0_. We use $\mu_{-}^{0}>\mu_{+}^{0}$ to describe the fact that anions are more difficult to enter into the SEI [23], as shown in Figure 2b.

For kinetic parameters, *D*_bulk_ is usually on the order of 10^−11^ m^2^ s^−1^ according to ref. [74]. $D_{\mathrm{SEI}}^{\mathrm{inner}}$ is several orders lower than *D*_bulk_[75]. $D_{\mathrm{SEI}}^{\mathrm{por}}$ is approximated using the Bruggeman relationship [35]. Here we use *D*_bulk_ =  1 × 10^−11^ m^2^ s^−1^, $D_{\mathrm{SEI}}^{\mathrm{inner}}=1\times 10^{-13}\;m^{2}\;s^{-1}$ and $D_{\mathrm{SEI}}^{\mathrm{por}}=D_{\mathrm{bulk}}\;\varepsilon_{p}^{3/2}$.

For the reaction parameters, α is 0.5 as usual though we understand there is no fundamental reason for this specific choice [54, 76]. *k*_0_ is usually on the order of 10^−4^ mol m^−2^ s^−1^ according to ref. [77]. Here we use α  =  0.5 and *k*_0_ =  3 × 10^−4^ mol m^−2^ s^−1^ as basal values.

Frequencies in experiments usually range from MHz to µHz [69]. Here we set the frequency range from 50 MHz to 10 mHz. The upper limit of 50 MHz ensures that the complete impedance response of the bulk solution can be observed. The lower limit of 10 mHz is sufficient to observe the full details of the reaction and diffusion. *E*_M_ is the electrode potential referenced to the electrostatic potential in the bulk electrolyte solution, where an ideal RE is placed.

For the parameters used in the inner‐layer model, we set $D_{\mathrm{SEI}}^{\mathrm{por}}=D_{\mathrm{bulk}}$ and $\mu_{\pm }^{0,\mathrm{por}}=0$, while keeping other parameters the same as those in the two‐layer model.

Employing the two‐layer SEI model introduced in the section of the model development, we calculate the steady distributions of ϕ(x), *c*_+_(*x*) and *c*_−_(*x*) in the SEI at different porosities under ideally blocking conditions (see Figure S2). The distributions of ϕ(x), *c*_+_(*x*) and *c*_−_(*x*) exhibit two‐stage profiles in the SEI. Using the steady‐state distributions of ϕ(x) and *c*_±_(*x*) as initial values, we calculate the impedance response of the two‐layer SEI model and compare it with the results of the inner‐layer model (see Figure S3). The two‐layer SEI model and the inner‐layer SEI model overlap in the examined range of porosities, with slight differences observed in the low‐frequency range. Therefore, for the parameters used here, we conclude that the impedance of the SEI is predominantly determined by the inner layer. In the following calculations, the SEI specifically refers to its inner layer unless otherwise noted.

### 3.2 Steady Distributions of $\phi \left(x\right),\;c_{+}\left(x\right)$ and *c*_−_(*x*)

**FIGURE 4:**
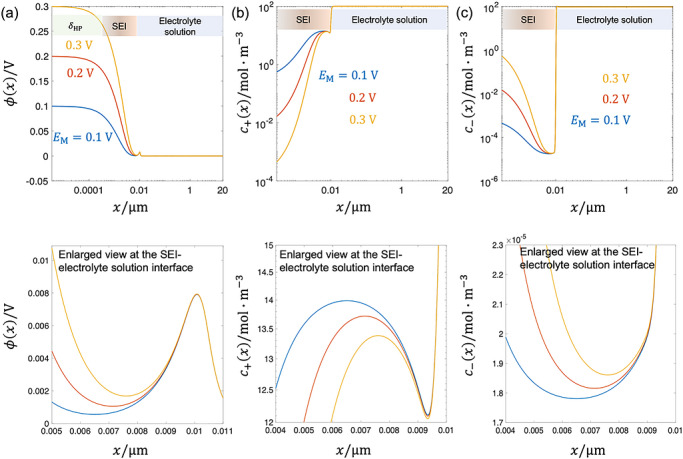
(a) Distribution of ϕ(x), (b) distribution of *c*_+_(*x*), and (c) distribution of *c*_−_(*x*) at different *E*_M_, respectively. Parameters used in the numerical calculation are as follows: $c_{\pm }^{\mathrm{bulk}}=0.1\;M$, *D*_bulk_ =  1 × 10^−11^ m^2^ s^−1^, *D*_SEI_ =  1 × 10^−13^ m^2^ s^−1^, $\mu_{-}^{0}=0.4\;\mathrm{eV},$ $\mu_{+}^{0}=0.05\;\mathrm{eV},$ ε_0_ =  8.85 × 10^−12^ F m^−1^, ε_S_ =  13ε_0_, ε_SEI_ =  6ε_0_, *d*_SEI_ =  10 nm, *x*_b_ =  20 µm.

Figure 4a–c presents the distributions of $\phi \left(x\right),\;c_{+}\left(x\right)$ and *c*_−_(*x*) with varying *E*_M_ respectively, using a logarithmic scale to illustrate the spatial distribution in the SEI. ϕ(x) shows a nonmonotonic profile consisting of decreasing, increasing, and then decreasing regions, with a pronounced peak at the interface between the SEI and the electrolyte solution. This nonmonotonic behavior arises from the presence of immobile background charge within the SEI. Without this consideration ($c_{\mathrm{back}}^{\mathrm{SEI}}=0$), the potential exhibits a monotonically decreasing or increasing trend depending on applied *E*_M_, see Figure S4a. As *E*_M_ becomes more positive, ϕ(x) within the SEI increases generally, while remaining unchanged outside. *c*_+_(*x*) adopts a non‐monotonic increasing‐decreasing‐increasing profile. As *E*_M_ becomes more positive, *c*_+_(*x*) within the SEI decreases due to the enhanced electrostatic repulsion. Conversely, *c*_−_(*x*) within the SEI increases due to the enhanced electrostatic attraction. However, changes in *c*_−_(*x*) are relatively small due to the higher standard chemical potential ($\mu_{-}^{0}$) of anions in the SEI, which makes their entry into the SEI more difficult.

### 3.3 Impedance of SEI under Ideally Blocking Conditions

**FIGURE 5:**
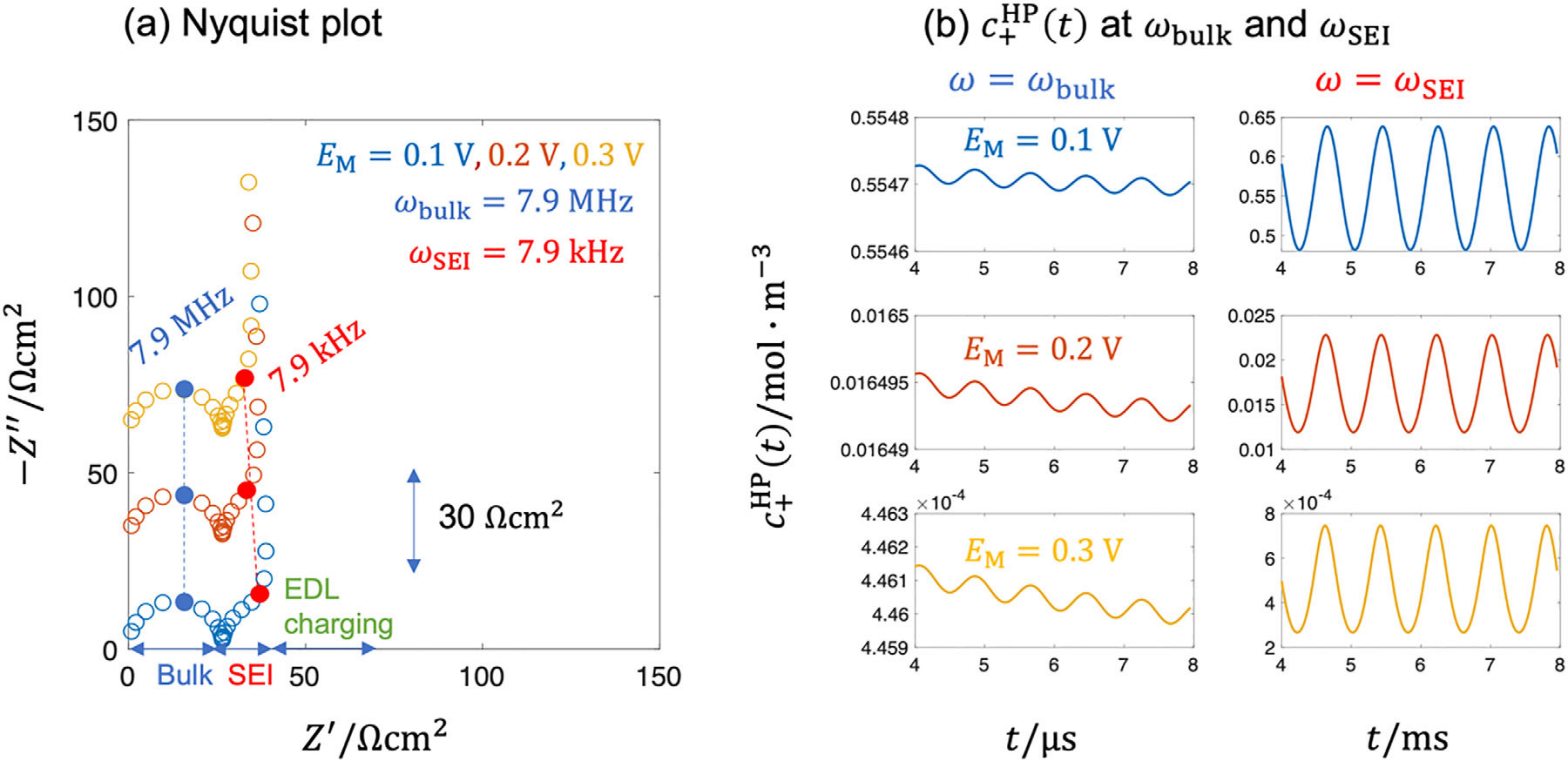
(a) Nyquist plot, (b) changes of $c_{+}^{\mathrm{HP}}\left(t\right)$ at ω_bulk_ and ω_SEI_ at different *E*_M_. Frequency ranges from 50 MHz to 500 Hz. Other parameters used in calculation are the same as those in Figure 4.

Using the steady‐state distributions of ϕ(x) and *c*_±_(*x*) in Figure 4 as initial values, we solve the dynamic PNP equations and calculate the SEI impedance response shown in Figure 5a. The Nyquist plot features two semicircles and a straight line. The physical processes corresponding to the two semicircles and the straight line can be determined from time constants or characteristic frequencies. The present cases have two characteristic frequencies, $\omega_{\mathrm{bulk}}=\frac{1}{R_{\mathrm{bulk}}C_{\mathrm{bulk}}}$ and $\omega_{\mathrm{SEI}}=\frac{1}{R_{\mathrm{SEI}}C_{\mathrm{SEI}}}$ with $R_{\mathrm{bulk}}=\frac{x_{b}RT}{F^{2}D_{\mathrm{bulk}}\sum c_{i}^{\mathrm{bulk}}}$, $R_{\mathrm{SEI}}=\frac{x_{b}RT}{F^{2}D_{\mathrm{SEI}}\sum c_{i}^{\mathrm{SEI}}}$ being the solution and SEI resistance, and $C_{\mathrm{bulk}}=\frac{\varepsilon_{S}}{x_{b}}$, $C_{\mathrm{SEI}}=\frac{\varepsilon_{\mathrm{SEI}}}{d_{\mathrm{SEI}}}$ being the solution and SEI capacitance. Then we mark ω_bulk_ and ω_SEI_ in the Nyquist plot, where ω_bulk_ is located at the high‐frequency semicircle while ω_SEI_ at the mid‐frequency semicircle. Therefore, we attribute the high‐frequency semicircle to the coupling of *R*_bulk_ and *C*_bulk_, while the mid‐frequency semicircle corresponds to the coupling of *R*_SEI_ and *C*_SEI_. For blocking conditions, the low‐frequency straight line is caused by double‐layer charging [59, 60, 62, 63, 68].

To help understand the relaxation processes, Figure 5b gives the last five‐period profiles of $c_{+}^{\mathrm{HP}}\left(t\right)$ at ω_bulk_ and ω_SEI_, respectively. At ω  = ω_bulk_ , the duration of ten periods is ≈8 µs, while at ω  = ω_SEI_ , it prolongates to ≈8 ms. Ion relaxation in the SEI is not fully developed at ω  = ω_bulk_ , so that $c_{+}^{\mathrm{HP}}\left(t\right)$ changes a little bit. At ω  = ω_SEI_ , ion relaxation in the bulk solution is fully developed, so that $c_{+}^{\mathrm{HP}}\left(t\right)$ changes a lot. As *E*_M_ becomes more positive, $c_{+}^{\mathrm{HP}}\left(t\right)$ decreases due to the enhanced electrostatic repulsion.

### 3.4 Constant Phase Element Phenomenon at Charged SEI

**FIGURE 6:**
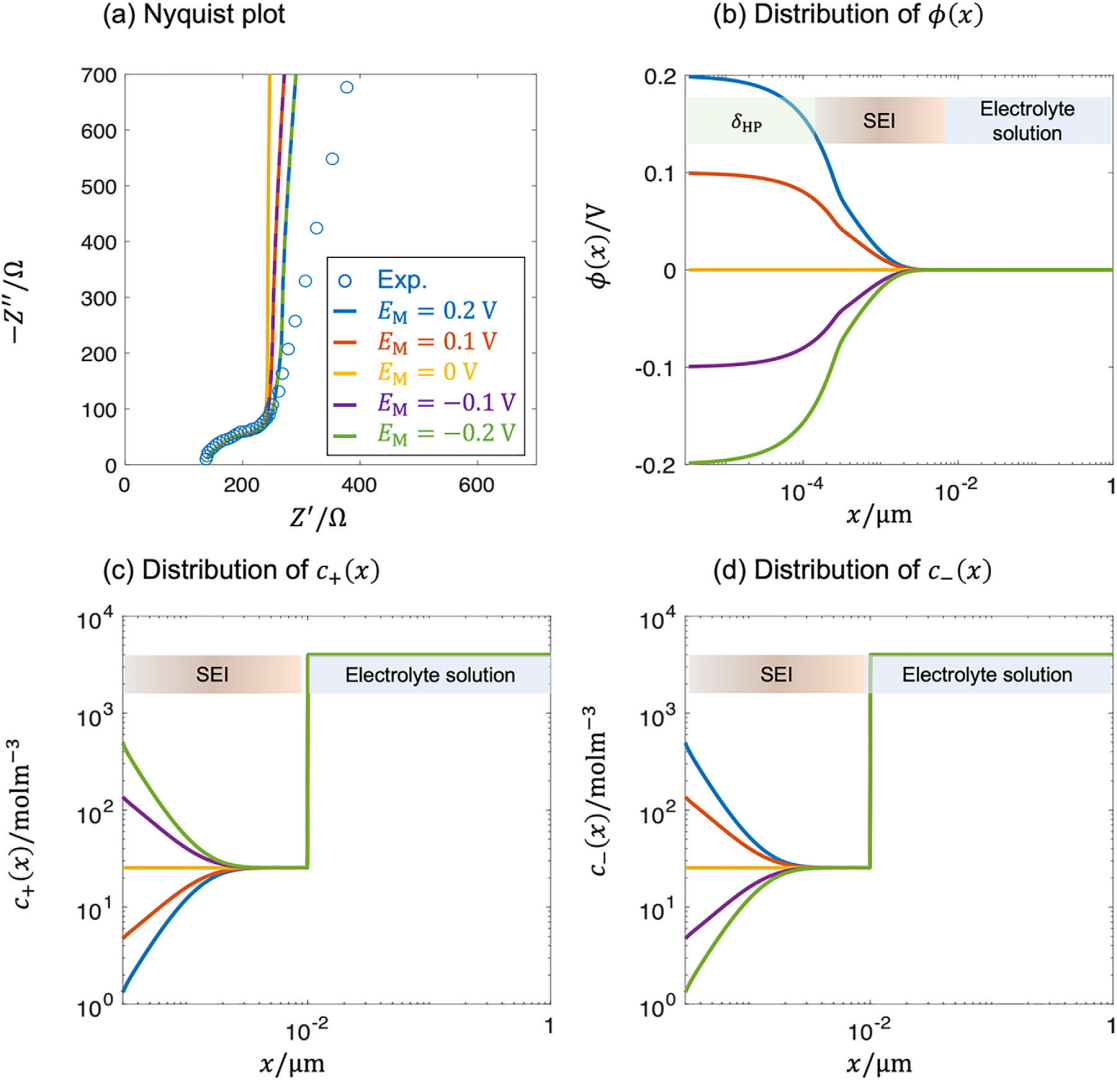
Comparison between the numerical solution of SEI impedance response and experiments. (a) Nyquist plot, (b) distribution of ϕ(x)/V, (c) distribution of *c*_+_(*x*)/mol m^−3^, (d) distribution of *c*_−_(*x*)/mol m^−3^. Parameters used in the numerical calculation are as follows: $D_{+}^{\mathrm{SEI}}=4\times 10^{-14}\;m^{2}\;s^{-1}$, $D_{-}^{\mathrm{SEI}}=D_{+}^{\mathrm{SEI}}\;/100$, $D_{+}^{\mathrm{bulk}}=5\times 10^{-11}\;m^{2}\;s^{-1}$, $D_{-}^{\mathrm{bulk}}=D_{+}^{\mathrm{bulk}}\;/100$, ε_S_ =  7.2ε_0_, *x*_b_ =  1 µm, *c*_SEI_ =  25.4 mol m^−3^, *d*_SEI_ =  10 nm. $c_{\pm }^{\mathrm{bulk}}=4\;m$ and frequency range (35 kHz–100 mHz) are the same as experimental values. Experimental data were taken from the study by Zhou et al. [33].

In Figure 5, we have simulated the SEI impedance response with changing *E*_M_. In this section, we rationalize the numerical model by fitting experimental data. The experiment was measured by Zhou et al. using a three‐electrode system (WE: Cu, CE: Lithium/LiCoO_2_, RE: Ag/AgNO_3_, electrolyte solution: 4 m LiFSI (DME)) at OCV condition (*E*_WE_ = 0 V vs Li/Li^+^) [33]. The measured SEI impedance consists of a high‐frequency circle and a low‐frequency tilted line as shown in Figure 6a, The high‐frequency semicircle is attributed to the coupling of *R*_SEI_ and *C*_SEI_, while the physical origin of the low‐frequency tilted line, resembling the CPE phenomenon [78, 79], is unclear.

Using a small‐amplitude current excitation as input after the static calculation, we calculate the impedance response at a series of *E*_M_ from 0 to ± 0.2 V, as shown in Figure 6a, which are compared with experimental data. When *E*_M_ =  0, the Nyquist plot consists of a high‐frequency circle and a nearly vertical line in the low‐frequency range. With *E*_M_ changing from 0 to ± 0.2 V, the high‐frequency circle changes a little while the low‐frequency line becomes more tilt with an angle less than 90°. Besides, the impedance curves at positive and negative *E*_M_ with an equal magnitude overlap, in accordance with earlier studies [80]. The tilting phenomenon of the low‐frequency line at more positive potentials has been observed in our previous simulations [59] and experimental results conducted by Katayama et al. [31].

It should be noted that our model demonstrates that nonelectroneutrality inside the SEI represents one possible origin of the observed low‐frequency CPE behavior, but it is by no means the only explanation. CPE responses are widely reported in electrochemical systems and have also been attributed to factors such as surface roughness [81], structural heterogeneity [78], and distributed time constants [82]. Therefore, the mechanism proposed here should be viewed as a physically plausible and complementary interpretation, rather than an exclusive attribution of the low‐frequency CPE phenomenon.

To explain this potential dependence, we examine the spatial distributions of ϕ(x), *c*_+_(*x*) and *c*_−_(*x*) shown in Figure 6b–d at different *E*_M_. When *E*_M_ =  0, ϕ(x) is uniform. As *E*_M_ becomes more positive, a pronounced gradient in ϕ(x) develops across the HP and the SEI. Correspondingly, gradients in *c*_+_(*x*) and *c*_−_(*x*) are developed in the SEI to screen the electrode surface charge. Anions accumulate at the HP due to the electrostatic attraction while cations are repelled from the HP. The increase of *c*_+_(*x*) and *c*_−_(*x*) causes the low‐frequency line to be more tilted, resembling “Warburg‐like behavior” associated with concentration polarization in the SEI. As *E*_M_ becomes more negative, cations accumulate at the HP while anions are repelled from the surface.

The effect of $D_{+}^{\mathrm{SEI}}$ on the SEI impedance is also investigated (see Figure S5). The high‐frequency semicircle representing the SEI impedance decreases with increasing $D_{+}^{\mathrm{SEI}}$.

### 3.5 Impedance of SEI with Lithium Plating and Stripping Reactions

In this section, we first introduce the steady distributions of $\phi \left(x\right),\;c_{+}\left(x\right)$, *c*_−_(*x*) and the net charge density *Q*_net_(*x*) in the presence of the Li plating and stripping reactions and then examine its impedance response.

#### 3.5.1 Steady Distributions of $\phi \left(x\right),\;c_{+}\left(x\right)$, *c*_−_(*x*) and *Q*_net_(*x*)

**FIGURE 7:**
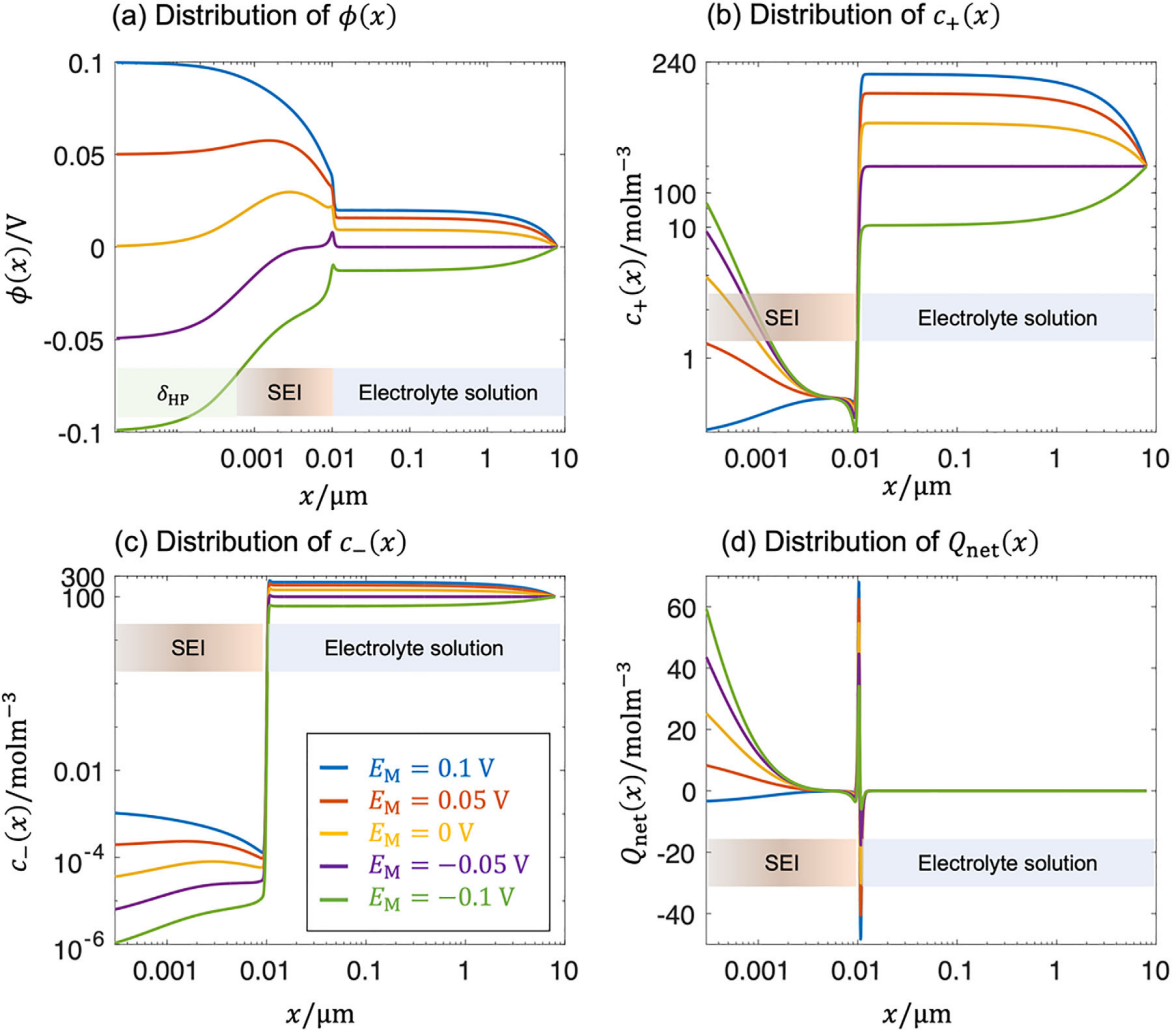
Internal properties of the SEI during plating and stripping reactions, represented as distributions of (a) ϕ(x), (b) *c*_+_(*x*), (c) *c*_−_(*x*), and (d) $Q_{\mathrm{net}}\;\left(x\right)=c_{+}\;\left(x\right)-c_{-}\left(x\right)-c_{\mathrm{back}}^{\mathrm{SEI}}\left(x\right)$. Parameters used in calcualtion are as follows: *k*_0_ =  3 × 10^−4^ mol m^2^s^−1^, *x*_b_ =  8 µm, *E*_eq_ =  0. Other parameters used in calculation are the same as those in Figure 4.

Using the numerical flowchart in Figure 3, we calculate the distributions of ϕ(x), *c*_+_(*x*), *c*_−_(*x*), and the net charge density $Q_{\mathrm{net}}\;\left(x\right)=c_{+}\;\left(x\right)-c_{-}\left(x\right)-c_{\mathrm{back}}^{\mathrm{SEI}}\left(x\right)$ at different electrode potentials, as shown in Figure 7. ϕ(x) shows a nonmonotonic profile at *E*_M_ < 0. As *E*_M_ becomes more positive, ϕ(x) shows a profile of increasing firstly, then decreasing to zero. When *E*_M_ is positive of 0.1 V, ϕ(x) follows a monotonically decreasing trend. According to the Poisson equation, the complex changing trends of ϕ(x) at different *E*_M_ are fundamentally governed by the distributions of *c*_+_(*x*), *c*_−_(*x*) and *Q*_net_(*x*). *c*_+_(*x*) at the HP decreases with more positive *E*_M_ due to the electrostatic repulsion. At the inner SEI/electrolyte solution interface, *c*_+_(*x*) shows a valley which decreases as *E*_M_ shifts negative due to greater consumption of cations. Outside the SEI, *c*_+_(*x*) increases at more positive *E*_M_, because the oxidation reaction products cations. *c*_−_(*x*) increases in the SEI and electrolyte solution with more positive *E*_M_ due to electrostatic attraction. Figure 7d shows the distribution of the net charge density *Q*_net_(*x*) at different *E*_M_. *Q*_net_(*x*) adopts a similar trend as *c*_+_(*x*) within the SEI. At the SEI‐electrolyte interface, *Q*_net_ shows a pair of peak and valley firstly, then increases to zero. In the electrolyte solution, *Q*_net_ is always zero which corresponds to electroneutrality conditions.

#### 3.5.2 Nyquist Plot at Different *E*_M_

**FIGURE 8:**
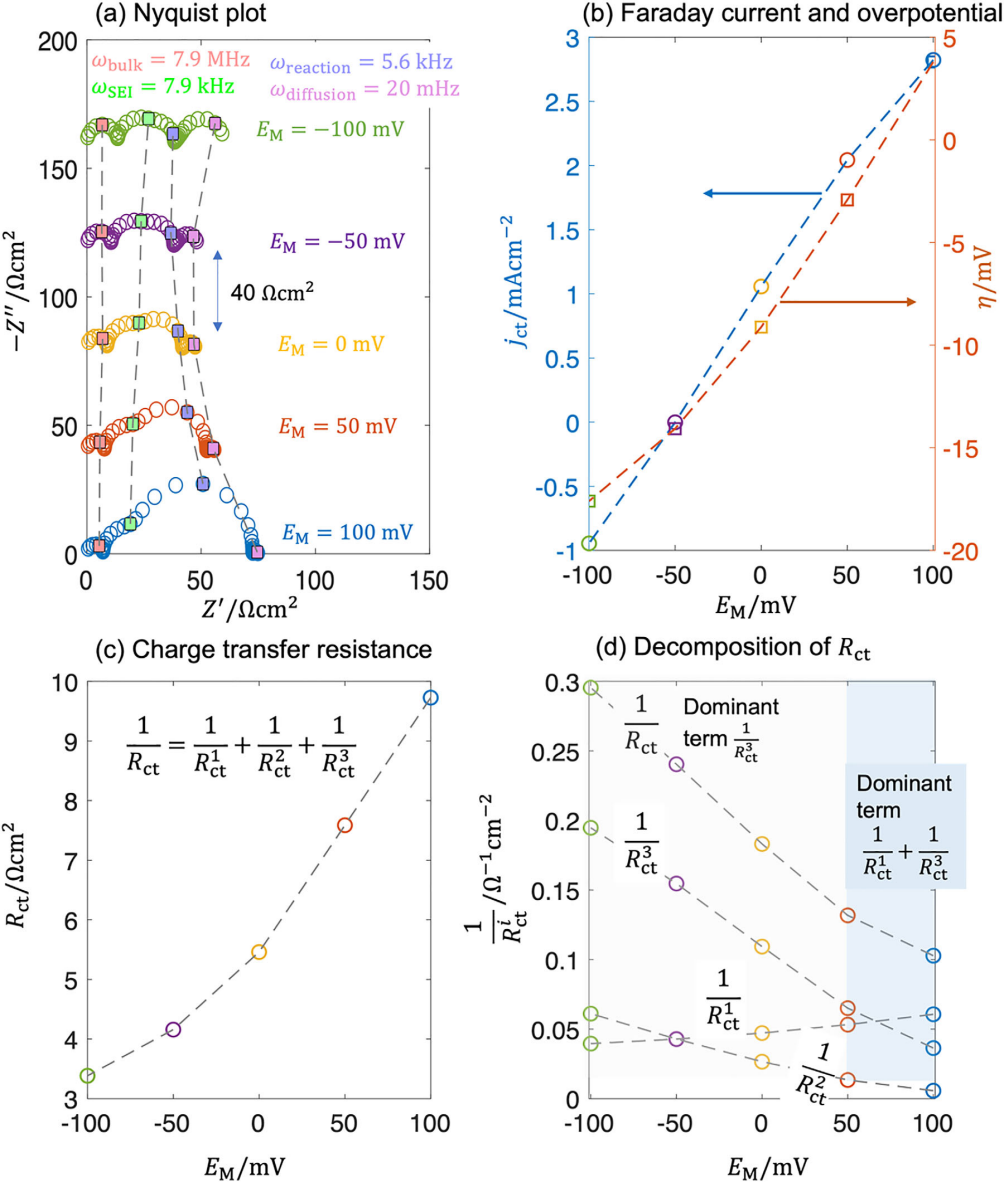
(a) Nyquist plot, (b) Faraday current density and overpotential, (c) charge transfer resistance *R*_ct_, and (d) decomposition of *R*_ct_ at different *E*_M_. Frequency ranges from 50 MHz to10 mHz. Other parameters used in the numerical calculation are the same as those in Figure 7.

Figure 8a shows the Nyquist plot of the SEI with the plating and stripping reactions at different *E*_M_. The Nyquist plot features four semicircles, with the first two semicircles corresponding to the bulk solution and the SEI as in Figure 5. The third and fourth semicircles are usually associated with reaction and diffusion [70]. We estimate the characteristic frequencies of reaction and diffusion, $\omega_{\mathrm{reaction}}=\frac{1}{R_{\mathrm{ct}}^{\mathrm{eq}}C_{\mathrm{dl}}}$ with $R_{\mathrm{ct}}^{\mathrm{eq}}$ being the charge transfer resistance at equilibrium conditions estimated from $R_{\mathrm{ct}}^{\mathrm{eq}}\approx \frac{RT}{Fk_{0}^{2}}$ [83] and *C*_dl_ being the EDL capacitance which is typically around 20 µF cm^−2^ [84], and $\omega_{\mathrm{diffusion}}=\frac{D_{\mathrm{bulk}}}{x_{b}^{2}}$ [85], respectively. Then we mark ω_reaction_ and ω_diffusion_ in the Nyquist plot. ω_reaction_ is located at the third semicircle while ω_diffusion_ at the fourth semicircle. Therefore, we attribute the third semicircle to the coupling of *R*_ct_ and *C*_dl_, while the fourth semicircle corresponds to the finite‐length diffusion. At more positive *E*_M_, the third semicircle grows while the fourth semicircle shrinks. The evolution of diffusion resistance can be interpreted from *c*_+_(*x*) shown in Figure 7b. As *E*_M_ becomes more positive, *c*_+_(*x*) increases in the electrolyte solution above its bulk value, thus decreasing the diffusion resistance. Figure 8a confirms this interpretation, showing a consistent decrease in the magnitude of the diffusion impedance.

To understand the changing trend of *R*_ct_, we calculate the Faraday current density and overpotential shown in Figure 8b. At more positive *E*_M_, *j*_ct_ changes from negative to positive values, namely, the net reaction transitions from reduction to oxidation reaction. η shows a similar trend from negative to positive values, driving the reaction transitioning from reduction to oxidation. However, *j*_ct_ does not always follow η. Specially, the oxidation reaction can occur even at negative η. The discrepancy originates from the fact that the SEI is charged, which causes a nonuniform distribution of ϕ(x) even at *E*_M_ =  0 in Figure 7a. Further, we derive *R*_ct_ by linearizing the Equation (9),

(15) $$R_{\mathrm{ct}}=\frac{R_{\mathrm{ct}}^{\mathrm{eq}}}{\alpha e^{\frac{\alpha \eta }{RT}}+\left(1-\alpha \right)\frac{c_{+}^{\mathrm{HP}}}{c_{+}^{0}}e^{-\frac{\left(1-\alpha \right)F}{RT}\eta }-\frac{d\left(c_{+}^{\mathrm{HP}}/c_{+}^{0}\right)}{d\left(\eta F/RT\right)}e^{-\frac{\left(1-\alpha \right)F}{RT}\eta }}\;$$

The changing trend of *R*_ct_ versus *E*_M_ is presented in Figure 8c, which is consistent with that observed in Nyquist plot in Figure 8a but there are quantitative deviations. The reason is that η used in Equation (15) is a collective variable including contributions from kinetic polarization, mass transport and ohmic resistance, resulting a smaller *R*_ct_ compared with that obtained from the Nyquist plot.

To further analyze the dependence *R*_ct_ on *E*_M_, we decompose it into three terms with its reciprocal form,

(16) $$\begin{array}{c} \frac{1}{R_{\mathrm{ct}}}=\frac{1}{R_{\mathrm{ct}}^{1}}+\frac{1}{R_{\mathrm{ct}}^{2}}+\frac{1}{R_{\mathrm{ct}}^{3}}\\ \frac{1}{R_{\mathrm{ct}}^{1}}=\frac{\alpha e^{\frac{\alpha \eta }{RT}}}{R_{\mathrm{ct}}^{\mathrm{eq}}}\\ \frac{1}{R_{\mathrm{ct}}^{2}}=\frac{\left(1-\alpha \right)\frac{c_{+}^{\mathrm{HP}}}{c_{+}^{0}}e^{-\frac{\left(1-\alpha \right)F}{RT}\eta }}{R_{\mathrm{ct}}^{\mathrm{eq}}}\\ \frac{1}{R_{\mathrm{ct}}^{3}}=-\frac{\frac{d\left(c_{+}^{\mathrm{HP}}/c_{+}^{0}\right)}{d\left(\eta F/RT\right)}e^{-\frac{\left(1-\alpha \right)F}{RT}\eta }}{R_{\mathrm{ct}}^{\mathrm{eq}}} \end{array}$$

Figure 8d presents the evolution of components of *R*_ct_ at different *E*_M_. We notice that $\frac{1}{R_{\mathrm{ct}}^{3}}$ is a dominant term at more negative *E*_M_, which causes a monotonic decrease of *R*_ct_. As *E*_M_ becomes more positive, $\frac{1}{R_{\mathrm{ct}}^{1}}+\frac{1}{R_{\mathrm{ct}}^{3}}$ dominates *R*_ct_. When *E*_M_ shifts from 50 to 100 mV, *R*_ct_ also increases which contradicts with conventional wisdom that a larger η causes a smaller *R*_ct_. The reason is that $\frac{1}{R_{\mathrm{ct}}^{3}}$ decreases to a greater extent than the increase in $\frac{1}{R_{\mathrm{ct}}^{1}}$, causing an overall decrease in 1/*R*_ct_, thus an increase in *R*_ct_. This phenomenon highlights the importance of an accurate description of the effects of nonelectroneutral SEI on the Li plating and stripping reactions [86].

### 3.6 Porous Inner‐Layer SEI

In practical systems, the inner layer of the SEI may form a 3D porous structure with distributed pore size along the thickness direction [11, 12, 87]. Here, we consider a simple case with a uniform pore size. Due to the axial symmetry, the 3D pore structure can be reduced to a 2D model, significantly reducing the computational cost.

In this part, we first introduce the 2D axisymmetric structure and its impedance with varying pore length and radius. Afterwards, we develop an ECM to approximate these numerical results.

#### 3.6.1 2D Axisymmetric Structure and Impedance Response

**FIGURE 9:**
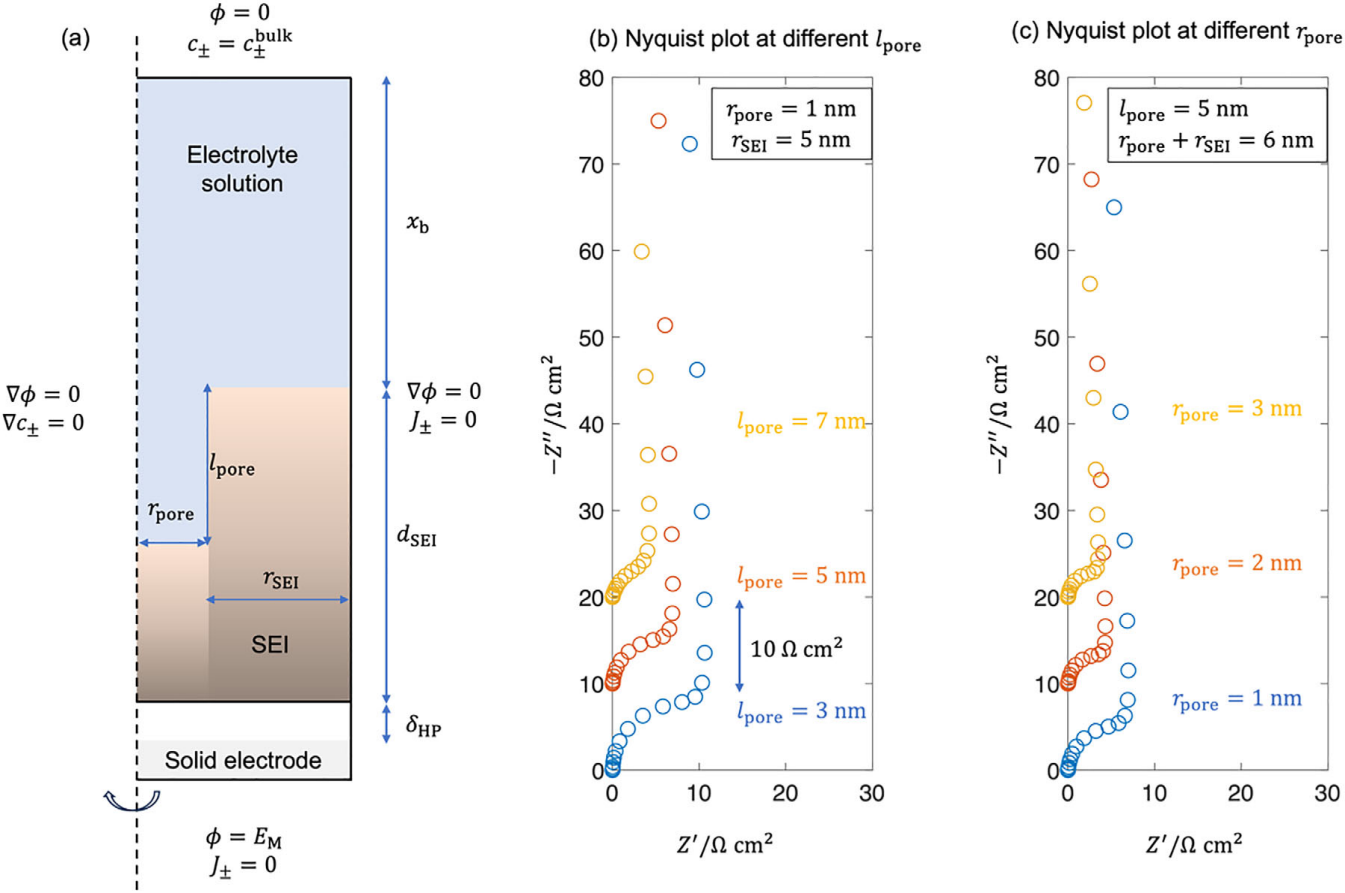
Porous inner‐layer SEI model. (a) 2D axisymmetric structure and boundary conditions, (b) Nyquist plot at different *l*_pore_ with fixed *r*_pore_ and *r*_SEI_, (c) Nyquist plot at different *r*_pore_ with fixed *l*_pore_ and *r*_pore_ + *r*_SEI_. Parameters used in calculation are as follows: *d*_SEI_ =  10 nm, *x*_b_ =  20 nm, other parameters are the same as those used in Figure 5.

The 2D axisymmetric structure of the inner‐layer SEI with micropores is shown in Figure 9a. Boundary conditions are as follows. The lower and upper boundary conditions, corresponding to the electrode surface and solution bulk, are the same as those in the previous 1D model under blocking conditions. For the right boundary, we assume the pore wall is blocking for both ions and electrons, namely, ∇ϕ=0 and *J*_±_ =  0. For the left boundary located at the axial line, the symmetry requires ∇ϕ=0 and ∇ *c*_±_ =  0. The impedance response of this 2D model can be reduced to the results of a 1D model when the inner‐layer SEI without pores, namely, *l*_pore_ =  0 (see Figure S6). With increasing pore radius *r*_pore_ and length *l*_pore_, the magnitude of the impedance decreases as shown in Figure 9b,c, respectively. It is intuitive that with larger *r*_pore_ and *l*_pore_, more electrolyte solution can percolate into the micropores, then the total impedance decreases. To understand it further, we develop an ECM to capture the evolution of total impedance with *r*_pore_ and *l*_pore_.

#### 3.6.2 ECM Model for Porous Inner‐Layer SEI

**FIGURE 10:**
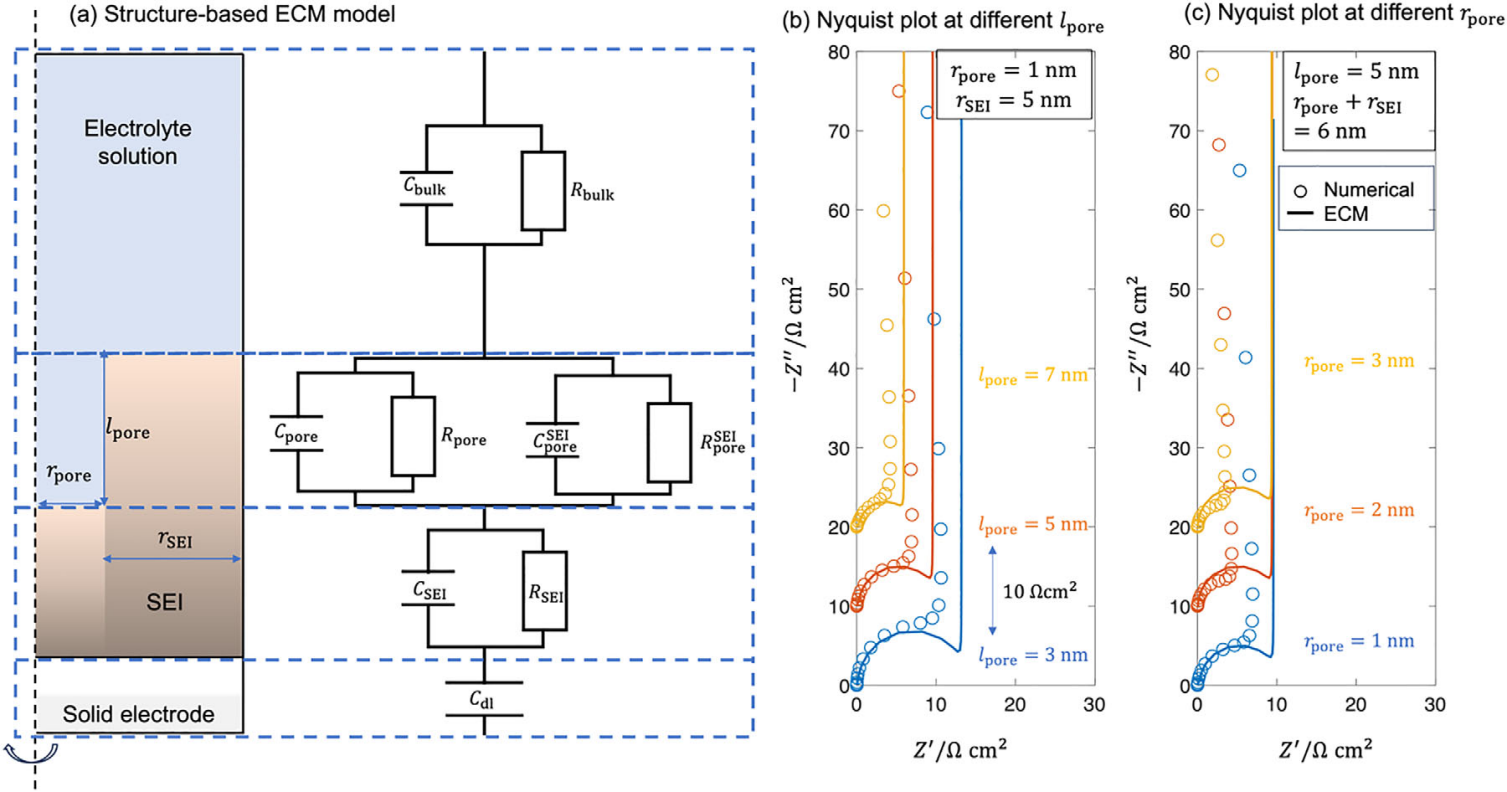
(a) Structure‐based ECM model. Comparison of Nyquist plots between numerical results calculated from the 2D axisymmetric model and the ECM model at different *l*_pore_ (b) and *r*_pore_ (c). Parameters used in the numerical calculation are the same as those used in Figure 9.

Here we develop a structure‐based ECM model to approximate the above numerical impedance results, as shown in Figure 10a. The porous SEI with electrolyte filled can be regarded as parallel channels of ion transport with different conductivities. Therefore, the mathematical expression of this ECM is given as,

(17) $$\begin{array}{c} Z_{\mathrm{tot}}=\frac{R_{\mathrm{bulk}}}{1+j\omega C_{\mathrm{bulk}}R_{\mathrm{bulk}}}+\frac{1}{\varepsilon_{\mathrm{pore}}/\;Z_{\mathrm{pore}}+\left(1-\varepsilon_{\mathrm{pore}}\right)/Z_{\mathrm{pore}}^{\mathrm{SEI}}}+\frac{R_{\mathrm{SEI}}}{1+j\omega C_{\mathrm{SEI}}R_{\mathrm{SEI}}}+\frac{1}{j\omega C_{\mathrm{dl}}}\\ Z_{\mathrm{pore}}=\frac{R_{\mathrm{pore}}}{1+j\omega R_{\mathrm{pore}}C_{\mathrm{pore}}}\\ Z_{\mathrm{pore}}^{\mathrm{SEI}}=\frac{R_{\mathrm{pore}}^{\mathrm{SEI}}}{1+j\omega R_{\mathrm{pore}}^{\mathrm{SEI}}C_{\mathrm{pore}}^{\mathrm{SEI}}} \end{array}$$

where *R*_bulk_/*R*_pore_/$R_{\mathrm{pore}}^{\mathrm{SEI}}$/*R*_SEI_ and *C*_bulk_/*C*_pore_/$C_{\mathrm{pore}}^{\mathrm{SEI}}$/*C*_SEI_ are the resistance and capacitance of the bulk electrolyte solution, the pore, the SEI nearby the pore and the SEI substrate, respectively. ε_pore_ is the porosity defined as,

(18) $$\varepsilon_{\mathrm{pore}}=\frac{\pi r_{\mathrm{pore}}^{2}}{\pi {\left(r_{\mathrm{pore}}+r_{\mathrm{SEI}}\right)}^{2}}\;$$

Comparison of Nyquist plots between numerical results calculated from the 2D axisymmetric model and the ECM in Equation (17) is shown in Figure 10b,c at different *l*_pore_ and *r*_pore_, respectively. The ECM model captures the trend of decreasing impedance with increasing *l*_pore_ shown in Figure 10b but fails to capture the evolution of decreasing impedance with increasing *r*_pore_. Specifically, *Z*_pore_ changes with *r*_pore_. Equation (17) neglects the interaction between the pore and the SEI near the pore, which can be supported by a theoretical analysis.

With the assumption of *D*_bulk_ ≫ *D*_SEI_, we obtain $Z_{\mathrm{pore}}\ll Z_{\mathrm{pore}}^{\mathrm{SEI}}$, then Equation (17) is reduced to,

(19) $$Z_{\mathrm{tot}}=\frac{R_{\mathrm{bulk}}}{1+j\omega C_{\mathrm{bulk}}R_{\mathrm{bulk}}}+\frac{1}{\varepsilon_{\mathrm{pore}}/\;Z_{\mathrm{pore}}}+\frac{R_{\mathrm{SEI}}}{1+j\omega C_{\mathrm{SEI}}R_{\mathrm{SEI}}}+\frac{1}{j\omega C_{\mathrm{dl}}}$$

When *Z*_pore_ ≪ *R*_SEI_, Equation (19) is reduced to,

(20) $$Z_{\mathrm{tot}}=\frac{R_{\mathrm{bulk}}}{1+j\omega C_{\mathrm{bulk}}R_{\mathrm{bulk}}}+\frac{R_{\mathrm{SEI}}}{1+j\omega C_{\mathrm{SEI}}R_{\mathrm{SEI}}}+\frac{1}{j\omega C_{\mathrm{dl}}}$$

Since that *R*_SEI_ is proportional to the thickness of the SEI substrate, *d*_SEI_ − *l*_pore_, *Z*_tot_ decreases with increasing *l*_pore_. However, *r*_pore_ is absent in Equation (20), explaining why *Z*_tot_ remains almost unchanged with increasing *r*_pore_.

A more detailed consideration requires accounting for nanoconfinement effects in the narrow pores of the inner‐layer SEI, where the dielectric response and ionic transport deviate from bulk behavior, making the pore impedance *Z*_pore_ dependent on the porosity and effective permittivity. Such effects are commonly described using transmission‐line models first proposed by De Levie [88] and further developed by Janssen [67, 89, 90], Gupta [91], Yaroshchuk [92], and also reviewed by Wu [93] and ourselves [35]. In the present work, these nanoconfinement effects are effectively included in our model through the distributed dielectric permittivity and pore radius, although a full transmission‐line treatment is beyond the scope of this article.

## 4 Discussion

In the section of results, we have developed a series of physical models considering complex physical and structural features of the SEI to understand its impedance response. We conclude that: (1) The SEI impedance is dominated by its inner layer; (2) the low‐frequency CPE behavior can be caused by nonuniform distributions of ion concentrations inside the SEI; (3) an unfavorable local reaction environment in the space charge layer will cause increasing charge transfer resistance with increasing overpotential; (4) simple ECM cannot capture the evolution of the SEI impedance with changing inner layer's pore radius.

In the following sections, we first formulate a physics‐based ECM from the numerical simulation and use it to extract parameters from experimental data at different operation conditions. Then collecting the physical insights from the planar and porous SEI models, we summarize key influencing factors of the SEI impedance.

### 4.1 Parameter Extraction from Experimental Data Using Physics‐Based ECMs

The numerical model under blocking conditions can be analytically solved at PZC (*E*_M_ = *E*_pzc_  =  0), using the same method developed in our previous work [59]. In this section, we use this analytical model to fit experimental data.

**FIGURE 11:**
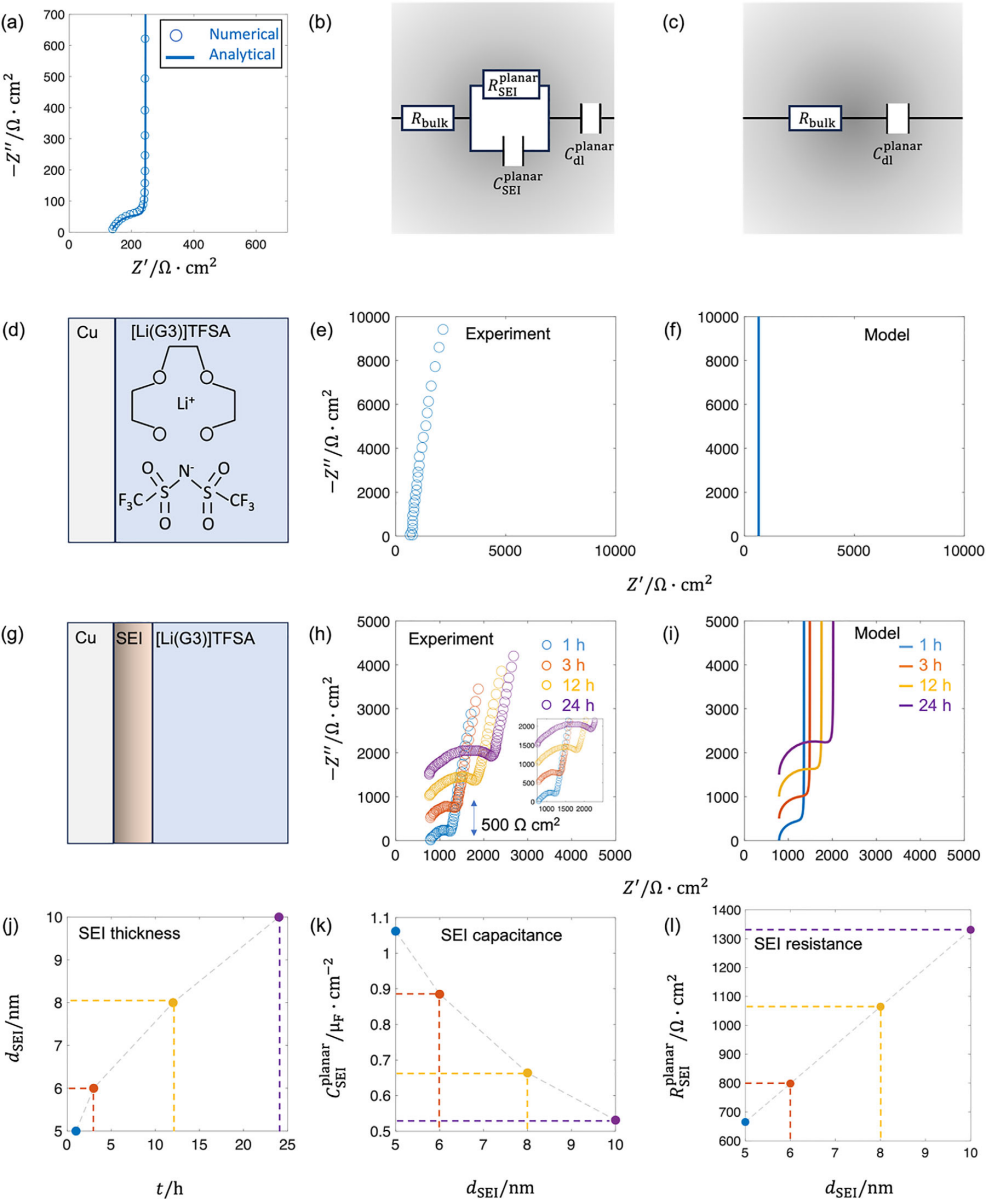
(a) Comparison between numerical and analytical solutions at PZC (The high‐frequency semicircle corresponding to the solution is neglected). (b) ECM after SEI formation. (c) ECM before SEI formation. (d) Schematic diagram before SEI formation. (e) Experimental data before SEI formation. (f) Model results before SEI formation. (g) Schematic diagram after SEI formation. (h) Experimental data after SEI formation. (i) Model results after SEI formation. (j) Fitted SEI thickness. (k) Fitted SEI capacitance. (l) Fitted SEI resistance. Experimental data were taken from the study by Serizawa et al. [94].

The analytical expression at PZC is as follows,

(21) $$\begin{array}{ccc} & & Z_{\mathrm{planar}}=R_{\mathrm{bulk}}+\frac{1}{j\omega C_{H}}\\ + & & \frac{1}{j\omega C_{\mathrm{GC}}^{0}}\frac{\frac{\tanh\left(\frac{d_{\mathrm{SEI}}}{\lambda_{D}}\sqrt{1+j\omega \lambda_{D}^{2}/D_{+}^{\mathrm{SEI}}}\right)}{\sqrt{1+j\omega \lambda_{D}^{2}/D_{+}^{\mathrm{SEI}}}}+j\omega d_{\mathrm{SEI}}\lambda_{D}/D_{+}^{\mathrm{SEI}}}{1+j\omega \lambda_{D}^{2}/D_{+}^{\mathrm{SEI}}} \end{array}$$

where *C*_H_ is the Helmholtz capacitance, $C_{\mathrm{GC}}^{0}$ the Gouy‐Chapman capacitance at PZC, and other parameters are defined in previous sections. We notice the experimental Nyquist plot in Figure 6a consists of a semicircle associated with SEI and a straight line. Therefore, the bulk solution is described using a pure resistance *R*_bulk_ neglecting the bulk dielectric capacitance *C*_bulk_ caused by its intrinsic dielectric response of the electrolyte solution in Equation (21). Figure 11a shows the agreement between numerical and analytical solutions.

As demonstrated in our previous work [59] as well as Barbero and Alexe‐Ionescu's work [62], asymptotic analysis is instrumental to understanding the cumbersome analytical expression *Z*_planar_. In the high‐frequency range, $\omega \gg \frac{D_{+}^{\mathrm{SEI}}}{d_{\mathrm{SEI}}^{2}}$, *Z*_planar_ is asymptotic to,

(22) $$Z_{\mathrm{planar}}\approx R_{\mathrm{bulk}}+\frac{1}{\frac{1}{R_{\mathrm{SEI}}^{\mathrm{planar}}}+j\omega C_{\mathrm{SEI}}^{\mathrm{planar}}}$$

where $R_{\mathrm{SEI}}^{\mathrm{planar}}=\frac{d_{\mathrm{SEI}}}{\sigma_{\mathrm{SEI}}}$ is the resistance of the SEI with $\sigma_{\mathrm{SEI}}=\frac{F^{2}\sum_{i=\pm }c_{i}^{\mathrm{SEI}}D_{i}^{\mathrm{SEI}}}{RT}$ being the total conductivity of the SEI, $C_{\mathrm{SEI}}^{\mathrm{planar}}=\frac{\varepsilon_{\mathrm{SEI}}}{d_{\mathrm{SEI}}}$ is the geometry capacitance of the SEI.

In the low‐frequency range, $\omega \ll \frac{D_{+}^{\mathrm{SEI}}}{\lambda_{D}^{2}}$, *Z*_planar_ is asymptotic to,

(23) $$\begin{array}{c} Z_{\mathrm{planar}}\approx R_{\mathrm{bulk}}+R_{\mathrm{SEI}}^{\mathrm{planar}}+\frac{1}{j\omega C_{H}}+\frac{1}{j\omega C_{\mathrm{GC}}^{0}}=R_{\mathrm{bulk}}+R_{\mathrm{SEI}}^{\mathrm{planar}}+\frac{1}{j\omega C_{\mathrm{dl}}^{\mathrm{planar}}}\\ \;\frac{1}{C_{\mathrm{dl}}^{\mathrm{planar}}}=\frac{1}{C_{H}}+\frac{1}{C_{\mathrm{GC}}^{0}} \end{array}$$

Combining high‐frequency and low‐frequency asymptotic analysis, we transform *Z*_planar_ expressed in Equation (21) to an ECM shown in Figure 11b. We term this as a physics‐based ECM. In the absence of an SEI, the ECM in Figure 11b can be further simplified to the one shown in Figure 11c.

One should be aware of the limitations of the analytical solution *Z*_planar_. It is obtained at the PZC, totally neglecting steady nonuniform distributions of ions in the SEI. Therefore, it misses the CPE‐like behavior in the low‐frequency range as shown in Figure 6a.

Serizawa et al. measured the impedance response on Cu electrode immersed in [Li(G3)]TFSA before and after the SEI formation [94], as shown in Figure 11d,g, respectively. The experimental details including (cell type, electrode prep, electrolyte composition, etc.) are reproduced in Note S7. The experimental results, shown in Figure 11e,h, reveal a tilted line in the Nyquist plot before the SEI formation and a high‐frequency semicircle followed by a low‐frequency tilted line after the SEI formation. The size of the high‐frequency semicircle, representing the SEI resistance, increases over time, as seen in Figure 11h.

We fit the experimental data with physics‐based ECMs in Figure 11b,c. Fitting results before and after the SEI formation are shown in Figure 11f,i, respectively. The overall trends of the model closely align with the experimental results, with the primary discrepancy observed at low frequencies: a vertical line in the model compared to a titled line usually called CPE effects in the experiments [78, 79]. In the last section, we used the numerical model to reveal that the low‐frequency tilted line could be associated with the heterogeneous steady distributions in the SEI.

Figure 11j–l present the fitted SEI thickness *d*_SEI_, capacitance *C*_SEI_, and resistance *R*_SEI_, respectively. Over time, *d*_SEI_ increases due to the continued electrolyte decomposition. Consequently, *C*_SEI_ decreases, while *R*_SEI_ increases. The main difference between our model and Serizawa et al.’s treatment is that they calculated *C*_SEI_ from the CPE element, $C_{\mathrm{SEI}}=T^{\frac{1}{p}}\;{R_{\mathrm{SEI}}}^{\frac{1-p}{p}}$ with *p* being the CPE index and *T* being the CPE constant, respectively. However, a direct connection between *p* and *T* and the internal physical processes in the SEI is missing. It should be noted that the fitted SEI resistance is consistent with SEI thickening; however, it cannot be uniquely attributed to thickness growth. Changes in composition, morphology (e.g., porosity), phase formation, or degradation processes may equally contribute to the observed impedance evolution.

Both numerical and analytical models are based on 1D planar assumption. However, experimental results demonstrate that practical SEI has some porous structures [87, 95, 96], which exhibit different impedance response [35, 88]. Therefore, in the next section, we develop a porous SEI model to study the influence of the porous structure on the impedance response.

### 4.2 Influencing Factors of SEI Impedance

**FIGURE 12:**
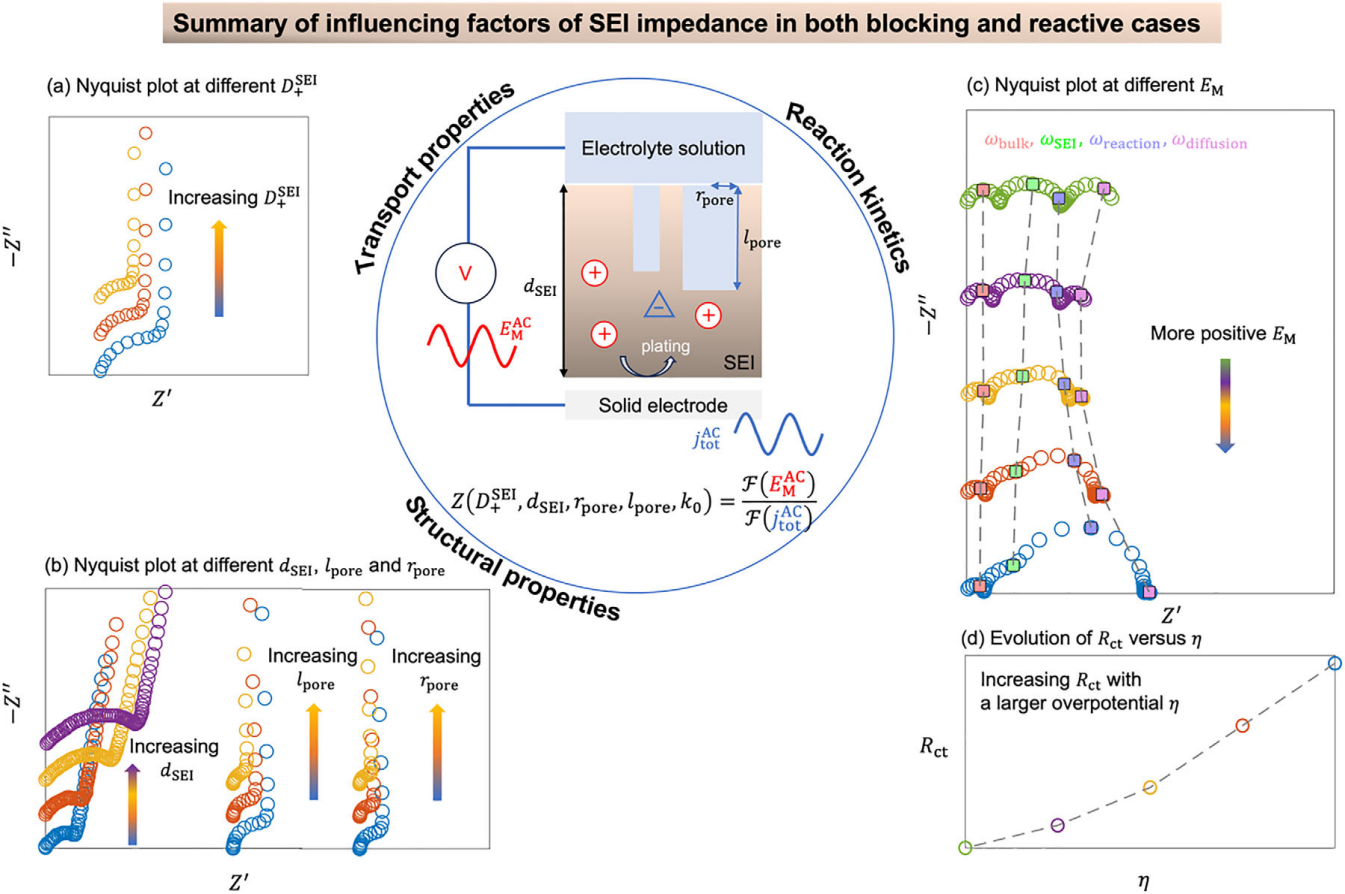
Summary of influencing factors of the SEI impedance in both blocking and reactive cases. (a) Nyquist plot at different $D_{+}^{\mathrm{SEI}}$, (b) Nyquist plot at different *d*_SEI_, *r*_pore_ and *l*_pore_ under blocking conditions. (c) Nyquist plot at different *E*_M_, (d) evolution of *R*_ct_ versus η in reactive cases.

In the previous section, we developed a series of numerical models—including the 1D two‐layer structural model, inner‐layer model in both blocking and reactive cases, and 2D porous inner‐layer model to investigate the SEI impedance response. We also formulated several approximate analytical solutions to assist in gaining physical insights. In this section, we summarize the factors influencing the SEI impedance. Figure 12 illustrates the effects of the cation diffusion coefficient $D_{+}^{\mathrm{SEI}}$, SEI thickness *d*_SEI_, pore length *l*_pore_ and pore radius *r*_pore_ on the SEI impedance response under blocking conditions and electrode potential *E*_M_ on the impedance response of the SEI with the lithium plating and stripping reactions. As $D_{+}^{\mathrm{SEI}}$, *r*_pore_, and *l*_pore_ increase, the SEI impedance decreases, while an increase *d*_SEI_ leads to an increase in the SEI impedance. As for the case of the SEI with the lithium plating and stripping reactions, the charge transfer resistance could grow, unexpectedly, with increasing overpotential due to an unfavorable local reaction environment.

In practical systems, transport parameters are often intrinsically correlated with structural characteristics and compositional effects of the SEI. In the present work, we do not explicitly resolve these correlations in detail; instead, we employ a simplified description based on the Bruggeman relationship to relate effective transport properties. Developing an impedance‐based workflow to determine the structural and transport parameters of the SEI is valuable and will be refined in future work.

In addition, we acknowledge that electrolyte chemistry and additives can substantially influence key model parameters, including ionic conductivity, transference number, dielectric properties, reaction kinetics, and SEI transport coefficients. These dependencies are not explicitly treated here. The present study aims to establish a general mechanistic modeling framework, whereas electrolyte‐specific parameterization will require dedicated experimental calibration and will be addressed in future investigations.

It should also be noted that the impedance response of SEI layers can often be described using classical equivalent‐circuit or transmission‐line models, which provide convenient phenomenological representations of experimental data. The objective of the present work, however, is not to demonstrate superior fitting performance compared to such models, but to develop a physically consistent continuum framework based on coupled ion transport and electrostatics. In contrast to equivalent‐circuit approaches, the present model explicitly relates the impedance response to spatial charge distributions, transport processes, and interfacial reactions within the SEI. Therefore, the framework should be viewed as complementary to circuit‐based analysis rather than a replacement, and a systematic comparison with simplified circuit representations is beyond the scope of the present study and will be addressed in the future work.

## 5 Conclusion and Outlook

This work presents a continuum theoretical framework for modeling internal processes and EIS response of the SEI in both blocking and reactive cases. The model framework is based on the Poisson‐Nernst‐Planck equations. A series of physical models including the 1D two‐layer SEI model, inner‐layer SEI model and 2D porous inner‐layer SEI model have been developed to account for practical complexities of the SEI.

The 1D model shows that the SEI impedance is predetermined by its inner layer. We then employed the 1D inner‐layer model to explain the shape change of EIS after the SEI formation. More importantly, the model gives rise to the low‐frequency CPE phenomenon, which only exists under nonelectroneutral conditions and is attributed to the heterogeneous distributions of concentration and electrostatic potential inside the SEI. We obtain an analytical solution to the impedance of SEI at the PZC (*E*_M_ = *E*_PZC_  =  0) and further develop a physics‐based equivalent circuit model (ECM) to extract key parameters of the SEI including thickness, capacitance and resistance from experimental EIS data.

Using the 2D porous inner‐layer SEI model, we analyzed the effects of the pore length and radius on the SEI impedance response and constructed a structure‐based ECM to approximate numerical results. This ECM captures the evolution of impedance changes with varying pore length but fails to reproduce the trend with varying pore radius. Constructing a transmission line model to describe the impedance changes with structural parameters will be a valuable work in the future.

In the impedance response of the SEI with lithium plating and stripping reactions, we find an unusual increase in charge transfer resistance of the stripping reaction with increasing overpotential caused by an unfavorable local reaction environment in the space charge layer, highlighting the importance of an accurate description of the effects of nonelectroneutral SEI on the lithium plating reaction. A future improvement of the model is to consider moving boundary conditions caused by the plating and stripping reactions and SEI growth, enabling operando modeling such as dynamic EIS response [97].

## Conflicts of Interest

The authors declare no conflicts of interest.

## Supporting information




**Supporting File**: advs75601‐sup‐0001‐SuppMat.docx.

## Data Availability

The data that support this study are available from the corresponding author upon reasonable request.
