# Supplementary material for: Impedance of Nonelectroneutral Solid Electrolyte Interphases With Nanopores: A Theoretical Model
Source: Adv Sci (Weinh). 2026 May 11;13(43):e75601. doi: 10.1002/advs.75601 (PMC13335918; doi:10.1002/advs.75601)
Supplement: Supplementary file 1 — Supporting File: advs75601‐sup‐0001‐SuppMat.docx. [file ADVS-13-e75601-s001.docx]

**Supporting information**

**Impedance of Nonelectroneutral Solid Electrolyte Interphases with Nanopores: A Theoretical Model**

*Chenkun Li^1,2^, Jun Huang^1,2*^*

*^1^Institute of Energy and Climate Research, IET-3: Theory and Computation of Energy Materials, Forschungszentrum Jülich GmbH, 52425 Jülich, Germany*

*^2^Faculty of Georesources and Materials Engineering, RWTH Aachen University, 52062 Aachen, Germany*

**Corresponding author:* [*ju.huang@fz-juelich.de*](mailto:ju.huang@fz-juelich.de)

## Note 1: comparison of impedance responses calculated from classical PNP equations and modified PNP equations

The controlling equations considering ion size effect, solvent polarization and short-range correlation above are as follows^1^,

| $\frac{\partial C_{i}}{\partial\tau}+\nabla J_{i}=0$  $J_{i}=-\frac{D_{i}}{D_{+}}\frac{2\lambda_{D}}{a}\left( C_{i}\left( 1-\gamma C \right) \right)\sinh\left[ \frac{a}{2\lambda_{D}}\left( \frac{1}{C_{i}}\frac{\partial C_{i}}{\partial X}+\frac{\gamma}{1-\gamma C}\frac{\partial C}{\partial X}\pm\frac{\partial U}{\partial X} \right) \right]$ | (S1) |
| --- | --- |
| $\frac{\partial^{2}U}{\partial X^{2}}+\frac{C_{+}-C_{-}}{2}=0$ | (S2) |

where $C_{i}$ is the dimensionless concentration referenced to its bulk solution $c_{i}^{0}$, *X* is the dimensionless spatial coordinate referenced to Debye length, $U$ is the dimensionless electric potential referenced to thermal voltage $RT/F$, $\tau=tD_{+}/\lambda_{D}^{2}$ is the dimensionless time, $D_{i}$ is the coefficient of species *i*, a is the ions size, $\gamma=a^{3}c_{0}N_{A}$ being the volume fraction in the bulk solution, $C=C_{+}+C_{-}$ being the total concentration.

As shown in Figure S1, the impedance response calculated from classical PNP equations and modified PNP equations considering ion size effect etc. has no change on shape while quantitative changes are observed. These results demonstrate that the classical PNP equations are sufficient to capture the primary features of impedance responses.

| 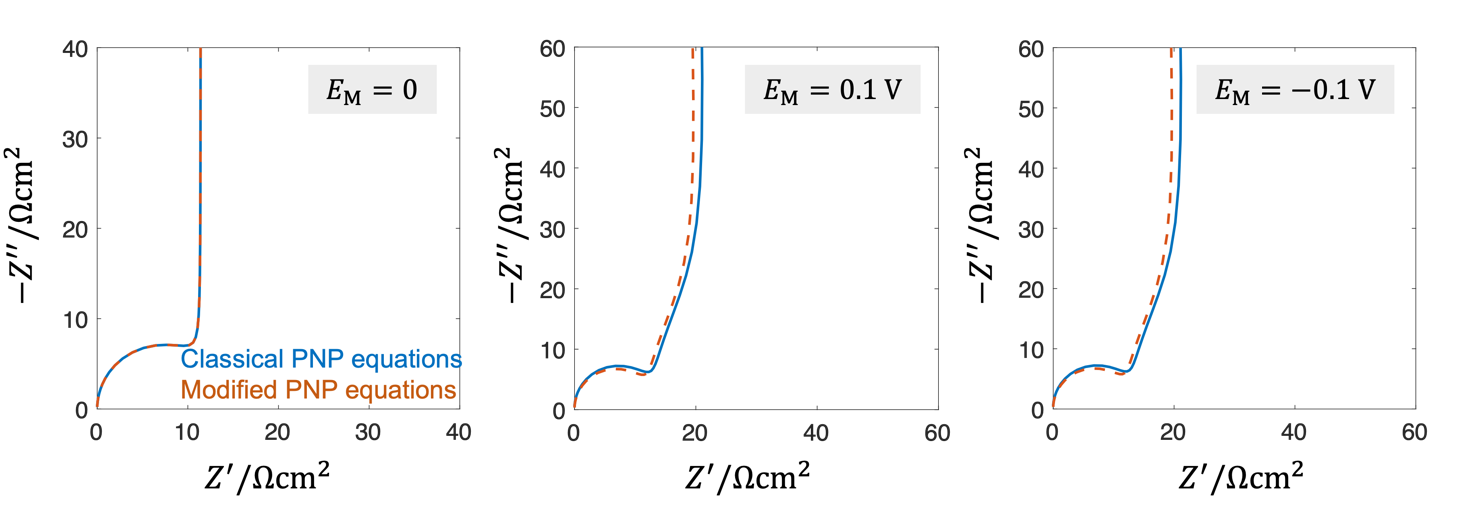 |
| --- |
| Figure S1 Comparison of impedance response calculated from classical PNP and modified PNP equations at different electrode potential. Parameters used in calculations are as follows: $a=3.5 Å$, $c_{0}=1\mathrm{mM}$, $D_{+}=D_{-}=1\times{10}^{-11} m^{2}s^{-1}$, $x_{b}=100\mathrm{nm}$, $\epsilon_{s}=78.5\epsilon_{0}$, frequency ranging from 10^6^ Hz to 100 Hz. |

## Note 2: steady distributions of $\boldsymbol{\phi}\mathbf{(}\boldsymbol{x}\mathbf{)}$ and $\boldsymbol{c}_{\boldsymbol{\pm}}\mathbf{(}\boldsymbol{x}\mathbf{)}$ with varying porosities calculated from two-layer and inner-layer SEI models

Figure S2 presents steady distributions of $\phi(x)$ and $c_{\pm}(x)$ with varying porosities calculated from the two-layer and inner-layer SEI models. Specifically, Figure S2 (a), (d), and (g) show the distributions of $\phi(x)$ at $E_{M}=0.1 V, 0,2 V, 0.3 V$, respectively. Figure S2 (b), (e) and (h) show the distributions of $c_{+}(x)$ at $E_{M}=0.1 V, 0,2 V, 0.3 V$. Figure S2 (c), (f) and (i) show the distributions of $c_{-}(x)$ at $E_{M}=0.1 V, 0,2 V, 0.3 V$. Two-stage profiles of $\phi(x)$ and $c_{\pm}(x)$ in the SEI are observed for the case of the two-layer SEI model. With the increasing porosity $\varepsilon_{p}$, the distributions of $\phi(x)$ and $c_{\pm}(x)$ almost have no changes. The distributions of $\phi(x)$ and $c_{\pm}(x)$ calculated from the two-layer and inner-layer SEI models are overlapped in the inner layer.

| 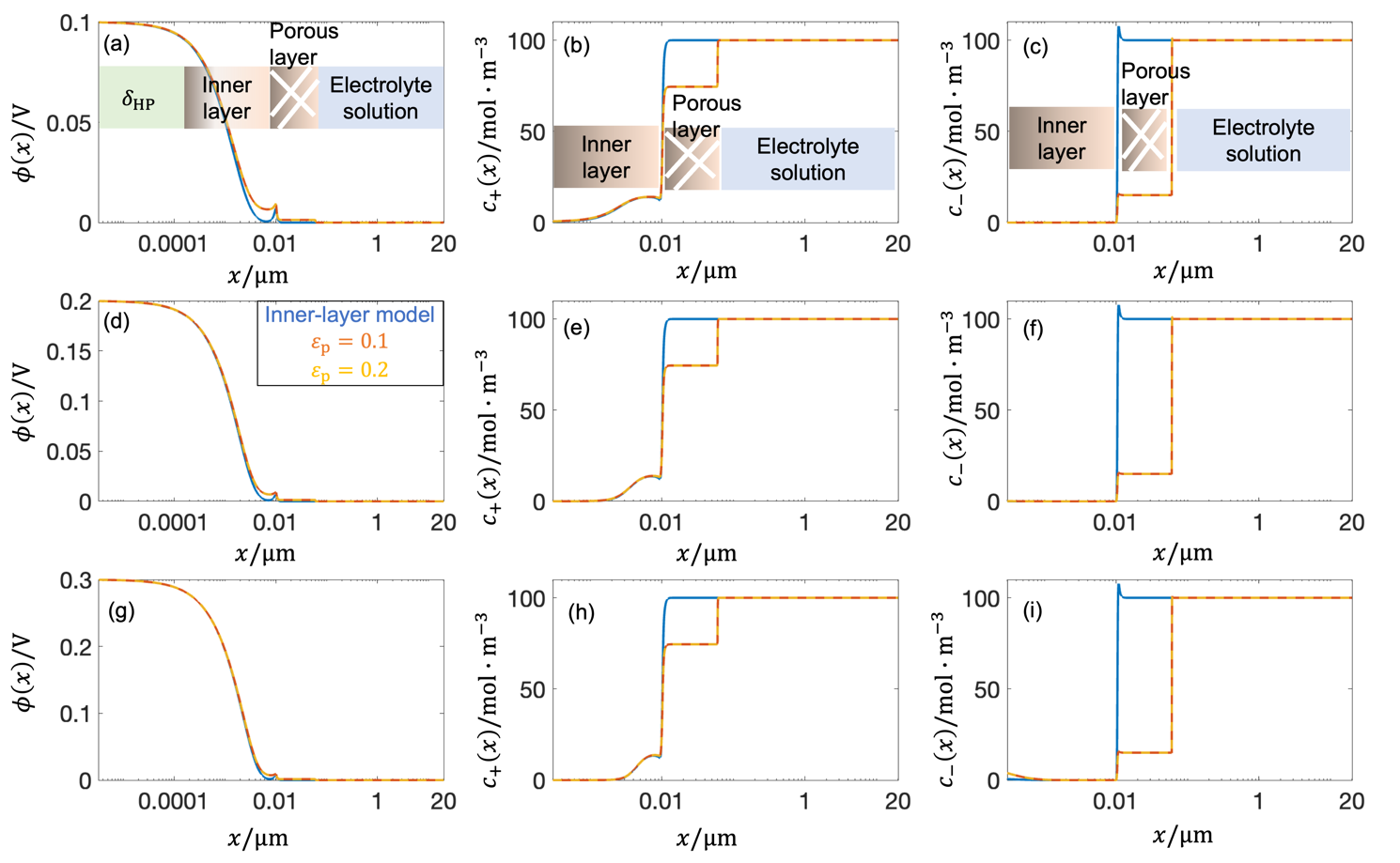 |
| --- |
| Figure S2 Steady distributions of $\phi(x)$ and $c_{\pm}(x)$ with varying porosity $\varepsilon_{p}$. (a), (b), (c), distributions of $\phi\left( x \right)$, $c_{+}(x)$ and $c_{-}(x)$ at $E_{M}=0.1 V$, (d), (e), (f), distributions of $\phi\left( x \right)$, $c_{+}(x)$ and $c_{-}(x)$ at $E_{M}=0.2 V$, (g), (h), (i), distributions of $\phi\left( x \right)$, $c_{+}(x)$ and $c_{-}(x)$ at $E_{M}=0.3 V$. Parameters used in calculation are as follows: $c_{\pm}^{\mathrm{bulk}}=0.1 M$, $\mu_{-}^{0,\mathrm{inner}}=0.4 \mathrm{eV},$ $\mu_{-}^{0,\mathrm{por}}=0.1 \mathrm{eV},$ $\mu_{+}^{0,\mathrm{inner}}=0.05 \mathrm{eV},$ $\mu_{+}^{0,por}=0.0125 \mathrm{eV}$, $d_{\mathrm{SEI}}^{\mathrm{inner}}=10 \mathrm{nm}$, $d_{\mathrm{SEI}}^{\mathrm{por}}=50 \mathrm{nm}$, $x_{b}=20 \mu m$. |

## Note 3: comparison of impedances calculated from two-layer and inner-layer SEI models

Figure S3 shows the comparison of impedances calculated from the two-layer and inner-layer SEI models at different porosities and $E_{M}$. The two-layer SEI model and the inner-layer SEI model exhibit overlapping behaviors in the investigating porosity range, with minor discrepancies noted in the low-frequency range. Therefore, for the parameters used here, we conclude the SEI impedance is dominated by the inner layer.

| 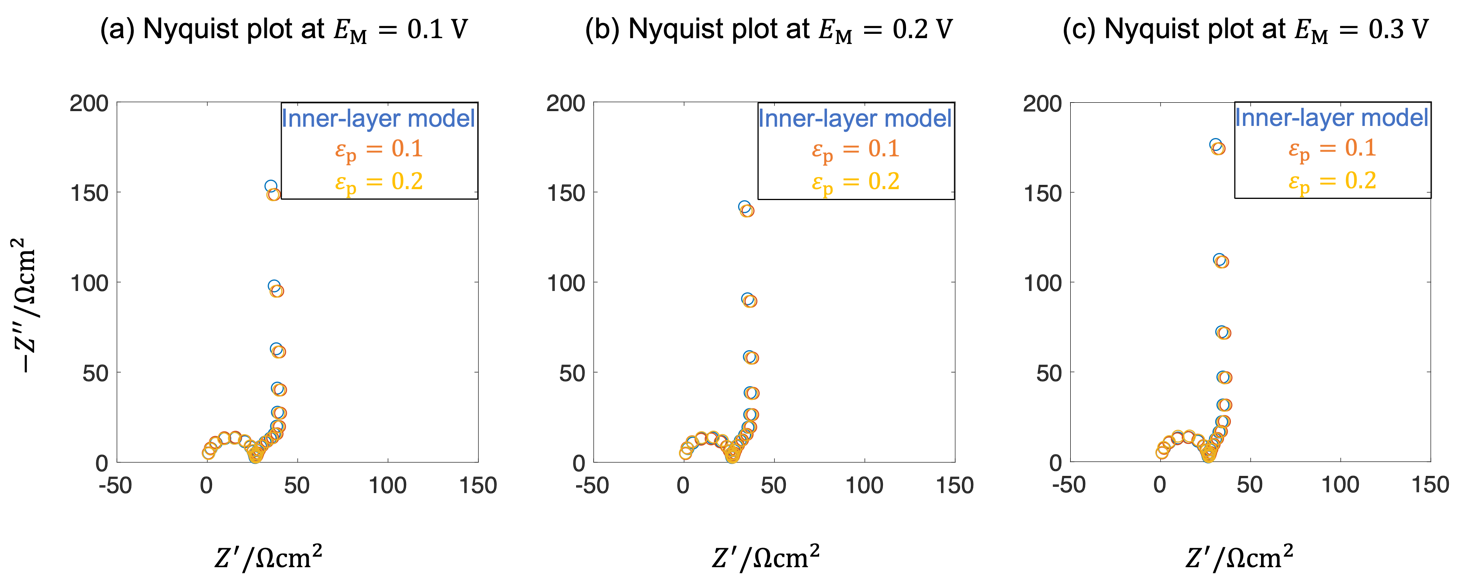 |
| --- |
| Figure S3 Comparison of impedances calculated from the two-layer and inner-layer SEI models. (a) Nyquist plot at $E_{M}=0.1 V$, (b) Nyquist plot at $E_{M}=0.2 V$, (c) Nyquist plot at $E_{M}=0.3 V$. Parameters used in calculation are as follows: $D_{\mathrm{bulk}}=1\times{10}^{-11} m^{2} s^{-1}$, $D_{\mathrm{SEI}}^{\mathrm{inner}}=1\times{10}^{-13} m^{2} s^{-1}$, $D_{\mathrm{SEI}}^{\mathrm{por}}=D_{\mathrm{bulk}}\varepsilon_{p}^{3/2}$. Frequency ranges from 50 MHz to 500 Hz. Other parameters used in calculation are the same as those in Figure S2. |

## Note 4: steady distributions of $\boldsymbol{\phi}\mathbf{(}\boldsymbol{x}\mathbf{)}$ and $\boldsymbol{c}_{\boldsymbol{i}}\mathbf{(}\boldsymbol{x}\mathbf{)}$ with varying $\boldsymbol{E}_{\mathbf{M}}$ for the case of $\boldsymbol{c}_{\mathbf{SEI}}^{\mathbf{back}}\boldsymbol{=0}$

Figure S4 shows the distributions of $\phi(x)$, $c_{+}(x)$ and $c_{-}(x)$ with varying $E_{M}$ for the case of $c_{\mathrm{SEI}}^{\mathrm{back}}=0$, respectively. $\phi(x)$ shows a monotonic decreasing profile along the SEI and electrolyte solution. As $E_{M}$ becomes more positive, $\phi\left( x \right)$ within the SEI increases generally, while remaining unchanged outside. $c_{+}(x)$ exhibits a monotonic increasing profile while $c_{-}(x)$ shows a non-monotonic decreasing-increasing profile. As $E_{M}$ becomes more positive, $c_{+}(x)$ within the SEI decreases due to the enhanced electrostatic repulsion, whereas $c_{-}(x)$ increases due to the enhanced electrostatic attraction.

| 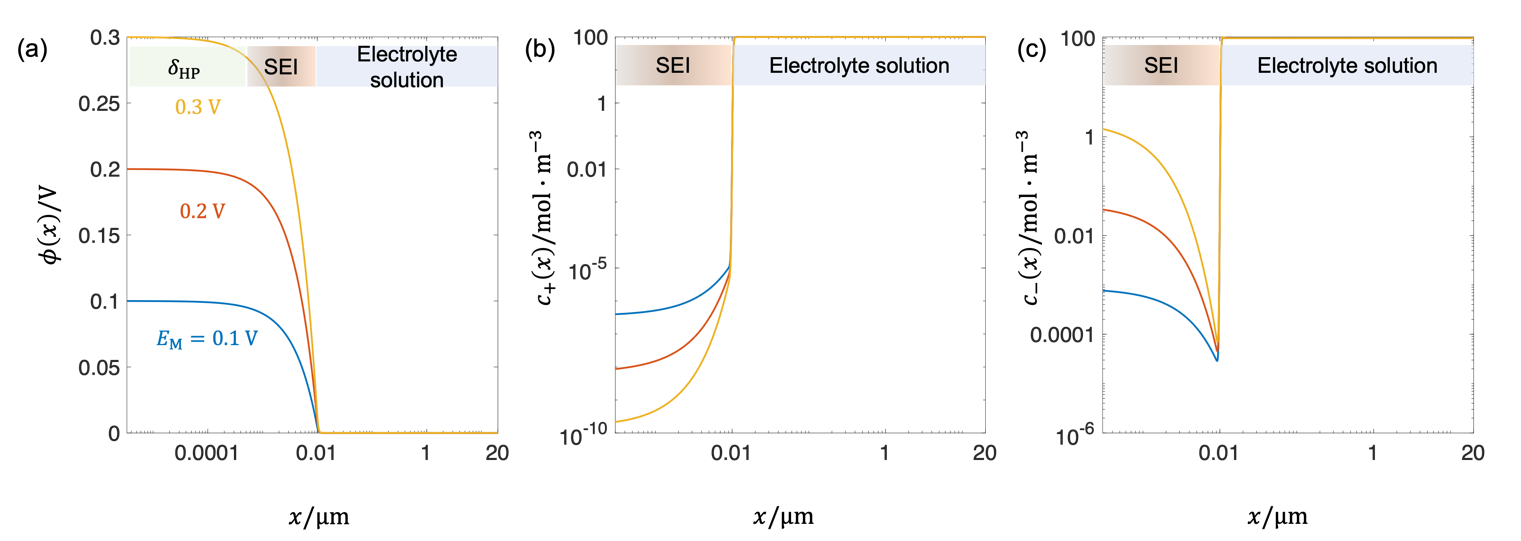 |
| --- |
| Figure S4 (a) Distribution of $\phi\left( x \right)$, (b) distribution of $c_{+}\left( x \right)$, and (c) distribution of $c_{-}\left( x \right)$ at different $E_{M}$, respectively. Parameters used in calculation are as follows: $\mu_{+}^{0}=\mu_{-}^{0}=0.4 \mathrm{eV}$. Other parameters are the same as those used in Figure 4 of the manuscript. |

## Note 5: effect of $\boldsymbol{D}_{\mathbf{+}}^{\mathbf{SEI}}$ on the SEI impedance response

Figure S5 compares the experimental results and numerical solutions of the SEI impedance at different $D_{+}^{\mathrm{SEI}}$. We notice that the high-frequency semicircle representing the SEI impedance decreases with increasing $D_{+}^{\mathrm{SEI}}$. It is intuitive that with higher $D_{+}^{\mathrm{SEI}}$, the SEI exhibits higher conductivity, then the impedance decreases.

| 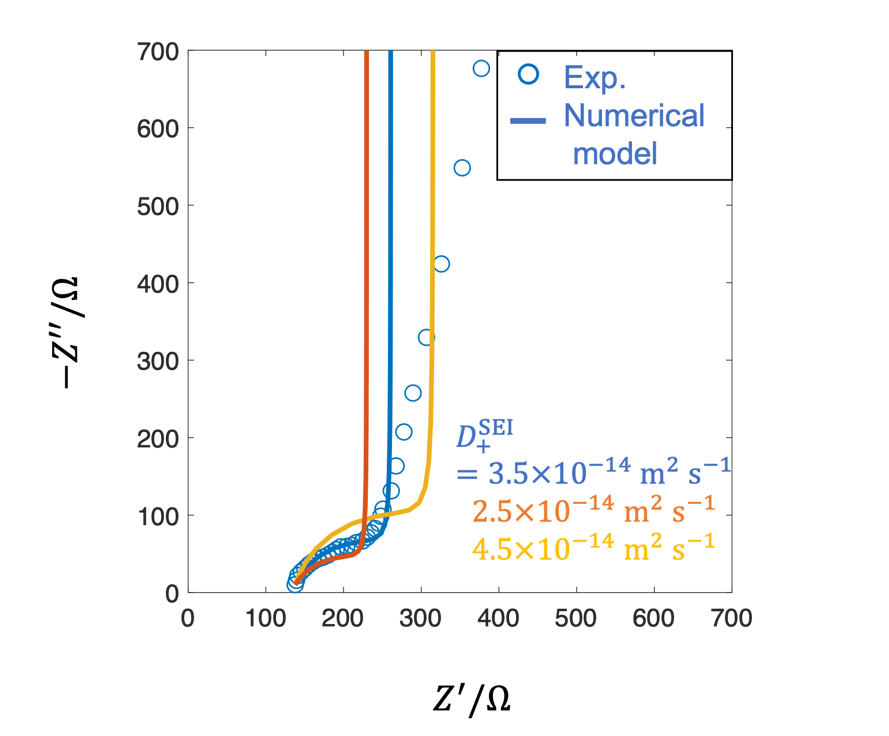 |
| --- |
| Figure S5 Comparison between experimental results and numerical solutions of the SEI impedance at different $D_{+}^{\mathrm{SEI}}$. $E_{M}=0$, other parameters are the same as those used in Figure 6 of the manuscript. |

## Note 6: validating the 2D axisymmetric model with 1D SEI model

Figure S6 compares the SEI impedances calculated from the 2D axisymmetric model and the 1D SEI model. The results calculated from the 2D axisymmetric model show good agreement with those from the 1D model for different values of the SEI thickness $d_{\mathrm{SEI}}$, thus indicating that the 2D model produces accurate results.

| 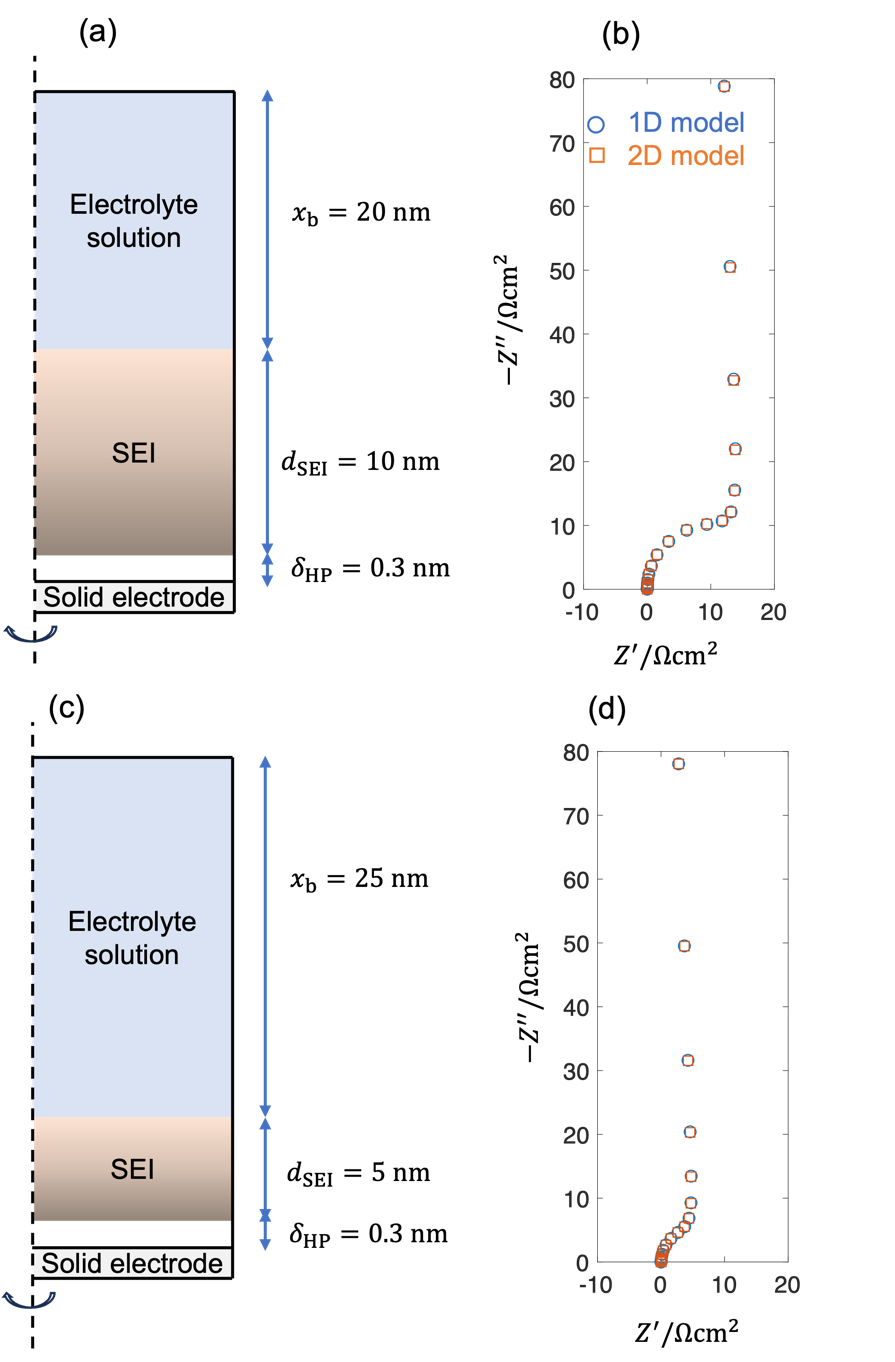 |
| --- |
| Figure S6 (a) 2D axisymmetric structure, (b) comparison of impedance response between 2D axisymmetric model and 1D SEI model with $d_{\mathrm{SEI}}=10 \mathrm{nm}$ and $x_{b}=20 \mathrm{nm}$, (c) 2D axisymmetric structure , (d) comparison of impedance response between 2D axisymmetric model and 1D SEI model with $d_{\mathrm{SEI}}=5 \mathrm{nm}$ and $x_{b}=25 \mathrm{nm}$. Parameters used in calculation are the same as those in Figure 9 of the manuscript. |

## Note 7: experimental details

Briefly, the electrolyte used in this study was an equimolar (50.0–50.0 mol%) solvate ionic liquid composed of lithium bis(trifluoromethanesulfonyl)amide (LiTFSA) and triglyme (G3), with water content below 60 ppm [2]. All electrolyte preparation and cell assembly were carried out in an Ar-filled glove box. Electrochemical measurements were performed in an airtight three-electrode cell at 298 K, using a Cu foil working electrode, and Li metal as both counter and reference electrodes. The Cu substrates were mechanically and electrochemically pretreated prior to use. SEI formation was conducted potentiostatically at 0 V vs. Li|Li⁺ for controlled durations. Electrochemical impedance spectroscopy (EIS), galvanostatic Li deposition/stripping tests, and coin-cell cycling measurements were performed to evaluate interfacial properties and reversibility.

## References

[1] Zhang, Z. M.; Gao, Y.; Chen, S. L.; Huang, J. Understanding Dynamics of Electrochemical Double Layers via a Modified Concentrated Solution Theory. *Journal of The Electrochemical Society* **2020**, *167*, 013519.

[2] Serizawa, N., Kitta, K., Tachikawa, N. and Katayama, Y., 2020. Characterization of the Solid-Electrolyte Interphase between a Cu Electrode and LiN (CF3SO2) 2-triglyme Solvate Ionic Liquid. *Journal of The Electrochemical Society*, *167*(11), p.110560.
